# Transition metals in angiogenesis – A narrative review

**DOI:** 10.1016/j.mtbio.2023.100757

**Published:** 2023-08-03

**Authors:** Johannes Dürig, Maurizio Calcagni, Johanna Buschmann

**Affiliations:** a University of Zürich, Faculty of Medicine, Pestalozzistrasse 3, 8032, Zurich, Switzerland; b University Hospital of Zürich, Department of Plastic Surgery and Hand Surgery, Rämistrasse 100, 8091, Zürich, Switzerland

**Keywords:** Transition metals, Angiogenesis inducing agents, Angiogenesis inhibitors, Vascular endothelial growth factors, Reactive oxygen species, Nanoparticles

## Abstract

The aim of this paper is to offer a narrative review of the literature regarding the influence of transition metals on angiogenesis, excluding lanthanides and actinides. To our knowledge there are not any reviews up to date offering such a summary, which inclined us to write this paper. Angiogenesis describes the process of blood vessel formation, which is an essential requirement for human growth and development. When the complex interplay between pro- and antiangiogenic mediators falls out of balance, angiogenesis can quickly become harmful. As it is so fundamental, both its inhibition and enhancement take part in various diseases, making it a target for therapeutic treatments. Current methods come with limitations, therefore, novel agents are constantly being researched, with metal agents offering promising results. Various transition metals have already been investigated in-depth, with studies indicating both pro- and antiangiogenic properties, respectively. The transition metals are being applied in various formulations, such as nanoparticles, complexes, or scaffold materials. Albeit the increasing attention this field is receiving, there remain many unanswered questions, mostly regarding the molecular mechanisms behind the observed effects. Notably, approximately half of all the transition metals have not yet been investigated regarding potential angiogenic effects. Considering the promising results which have already been established, it should be of great interest to begin investigating the remaining elements whilst also further analyzing the established effects.

## Graphical abstract

**Figure:**
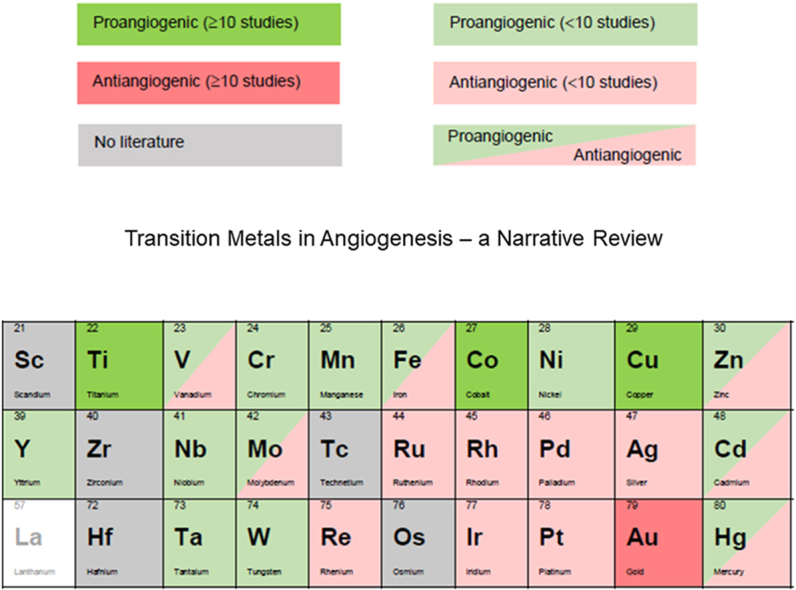

## Abbreviations

AGF: angiogenic growth factor
Akt: protein kinase B
ANG: angiopoietin
bFGF: basic fibroblast growth factor
BG: bioactive glass
BMSC: bone marrow stromal cell
CAM: chorioallantoic membrane
CdCl_2_: cadmium chloride
CD31: platelet endothelial cell adhesion molecule-1
Co^2+^: cobalt ion
CoCl_2_: cobalt chloride
COX: cyclooxygenase
Cr(VI): hexavalent chromium
CS: carbide nanosheet
CXCL8: chemokine ligand 8
diSe: diselenoether ligand
EC: endothelial cell
ECM: extracellular matrix
EGFL7: epidermal growth factor-like domain 7
EGFR: epidermal growth factor receptor
eNOS: endothelial nitric oxide synthase
EPC: endothelial progenitor cell
EPR: enhanced permeability and retention
ER: endoplasmic reticulum
ERK: extracellular signal-regulated kinase
FAC: ferric ammonium citrate
FAK: focal adhesion kinase
FGF: fibroblast growth factor
GC: glucocorticoid
GH: gelatin-hyaluronic acid hydrogel
gold-1a: gold (III) meso-tetraphenylporphyrin 1a
GR: glucocorticoid receptor
GSH: glutathione
hADSC: human adipose derived stem cell
Hb: hemoglobin
HIF1-α: hypoxia-inducible factor 1-alpha
HgCl_2_: mercury (II) chloride
HKa: cleaved high molecular weight kininogen
HMVEC: human microvascular endothelial cell
HNO: nitroxyl
H_2_O_2_: hydrogen peroxide
HS: heparin sulfate
HUVEC: human umbilical vein endothelial cell
IGF: insulin-like growth factor
IL: interleukin
iNOS: inducible nitric oxide synthase
LAC: lung adenocarcinoma cell
MAPK: mitogen-activated protein kinase
MEK: mitogen-activated protein kinase
MgO: magnesium oxide
miR: micro-RNA
MoO_3_: molybdenum trioxide
Nb-BG: niobium silicate bioactive glass
NBGS: niobium carbide nanosheet scaffolds
NF-κB: nuclear factor kappa-light-chain-enhancer of activated B cell
NGR: Asn-Gly-Arg
NiCl_2_: nickel chloride
NiO: nickel oxide
Ni_3_S_2_: nickel subsulfide
NiTi: nickel/titanium
NO: nitric oxide
NP: nanoparticle
NR: nanorods
PCL: poly-ε-caprolactone
Pd (II)-complex: palladium (II)-saccharinate complex of terpyridine
PEEK: polyether-ether-ketone
PERK: Protein kinase R-like endoplasmic reticulum kinase
PHD: prolyl hydroxylase
PIGF: placental growth factor
PI3K: phosphoinositide-3-kinase
PPC: polynuclear platinum complex
ps-TNC: iron-based nanocluster
PVP: polyvinylpyrrolidone
RCT: rhenium (I)-tricarbonyl-complex
RGD: Arg-Gly-Asp
ROS: reactive oxygen species
Ru (II)-8-hydroxyquinoline: PQ
[Ru^III^(N_2_O_2_)Cl_2_]Cl: Ru-1
SNC: silver nano-colloid
SPP: skin prefusion pressure
Sr: strontium
TGF-β: transforming growth factor-beta
Tie2: angiopoietin receptor
TIMP-1: tissue inhibitor of metalloproteinase-1
TiO_2_: titanium dioxide
TRAP: tartrate-resistant acid phosphatase
TTM: tetrathiomolybdate
VAC: organic vanadium salt
VC-III: vanadium (III)-l-cysteine
VE-cadherin: vascular endothelial cadherin
VEGF: vascular endothelial growth factor
VEGFR: vascular endothelial growth factor receptor
V_2_O_5_: vanadium pentoxide
VONR: vandadium oxide nanorod
VSMC: vascular smooth muscle cell
ZnO: zinc oxide
ZS/HA/Col: zinc silicate nano-hydroxyapatite/collagen

## 1 Introduction

Angiogenesis describes the formation of blood vessels by sprouting from existing vessels, a complex process which plays a crucial role in human physiology. Already starting during the early stages of embryogenesis, this process remains relevant all throughout human life ultimately enabling blood supply during tumorigenesis. Just as there are various healthy tissues dependent on blood vessels for the delivery of various nutrients, oxygen and immune cells, there are also various diseases dependent on the same processes. Depending on the disease in question, angiogenesis can be a desired effect or a detrimental process. Tumors for example, thrive on superfluous blood vessels, therefore, inhibition of angiogenesis would be desirable [1]. On the other hand, diabetes leads to destruction of existing vasculature [2]. In this case, promoting angiogenesis to produce new blood vessels would be the end goal. Indeed, understanding how to inhibit or enhance angiogenesis could lead to developing crucial therapeutic options in a clinical setting. There already exist methods to mediate angiogenesis as for example VEGF to promote angiogenesis; yet due to remaining limiting factors, these are not sufficient. Besides a short *in vivo* half-life of approximately half an hour, VEGF has been reported to induce vascular leakage and form disorganized blood vessels (and malformed lymphatic vessels as well) [3]. Consequently, alternatives should be explored.

Transition metals are essential for various bodily functions e.g., without iron, there would be no oxygen transport [4]. Cobalt, copper, and zinc belong to the essential trace elements within the human body [5]. Apart from their vital biological functions, transition metals are multipurpose elements used in various fields of medicine such as pharmaceutics, oncology, and implant materials. As a practical example, one can consider the production of such implant materials. Adequate angiogenesis is important for implants to successfully integrate within host tissues. Transition metals such as titanium are widely used to produce implants due to their ideal mechanical properties. It would be useful to have more extensive knowledge on angiogenic properties of these materials during the development of new implants, since choosing a proangiogenic component could further aid the tissue integration process. Indeed, the use of different elements could lead to materials with superior mechanical and biochemical properties. Furthermore, studies have indicated that implants can release particles into the peri-implant microenvironment and effect surroundings cells [6]. The release of proangiogenic particles could further enhance the vascularization process.

Some of the transition metals such as chromium and mercury can be found in our environment. Pollution in air, water and landfills is seen as a critical public health problem [7], as exposure has been linked to lung cancer and neurological damage [8]. The exact mechanisms of the cancerogenic effects have not been fully understood. As angiogenesis is to be considered one of the major processes required for tumor growth, it should be determined whether transition metals such as chromium owe their cancerogenic effects to the promotion of processes such as angiogenesis. Indeed, this knowledge would be paramount for the development of new anticancer drugs. By specifically targeting these proangiogenic particles, neovascularization and metastasis of solid tumors could be inhibited.

Metal based agents have been proven to display pro- and antiangiogenic properties, offering a potential alternative to current therapies. This specific research field remains in its beginning phases, as there is still much research needed before application in a clinical setting can be achieved. There have been review papers written on the role of inorganic metals in angiogenesis [[9], [10], [11]], however, as publications shed light on novel discoveries regularly, it is important to keep reviews updated. Furthermore, to our knowledge, there has not been a review conducted focusing solely on transition metals and their role in angiogenesis. This enticed us to write a narrative review, offering a summary of the current findings concerning the angiogenic potential of transition metals.

## 2 Methods

### 2.1 The purpose of review

There exist numerous studies investigating various metal elements and their effects on angiogenesis. Since this research field is novel and remains in its beginning stages, not many review papers have been written offering a summary of the current advancements. Furthermore, the increasing attention results in new studies being published regularly, therefore, updates are necessary. The aim of this paper is to offer an overview of the literature regarding the influence specifically of transition metals on angiogenesis. To our knowledge there are not any review papers up to date offering such a summary, which inclined us to write this narrative review.

### 2.2 Search methodology

The literature search was performed in the PubMed and Google Scholar databases. First, each transition metal was searched separately regarding its effects on angiogenesis with the use of key words from the MeSH database. As several transition metals have few publications concerning their angiogenic effects, MeSH terms were not used in such cases to allow a broader search.

### 2.3 Search strategy

Each element was searched in combination with the terms *angiogenesis*, *vascularization*, *proangiogenic* or *antiangiogenic*. If this method did not lead to any search results, the transition metals were searched in combination with other terms linked to angiogenesis, such as growth factors and other angiogenesis modulating agents. The references of the selected studies were also examined.

### 2.4 Inclusion and exclusion criteria

Articles published in peer reviewed journals regarding angiogenic effects of transition metals, in English, from 1970 through May 2023 were considered for this review. Studies analyzing the angiogenic effects of transition metals were included in this review; i.e. transition metals that directly activate key effectors of enhancing angiogenesis or inhibiting angiogenesis, with up- or downregulation of the main factors known to be involved in angiogenesis. Research that indicated angiogenic effects, which were not directly linked to a transition metal, were included in the discussion part but not included in the table summarizing each transition metal's direct pro- and antiangiogenic effects. Papers that did not specifically discuss angiogenesis or angiogenic related processes were excluded from this review.

**Fig. 1:**
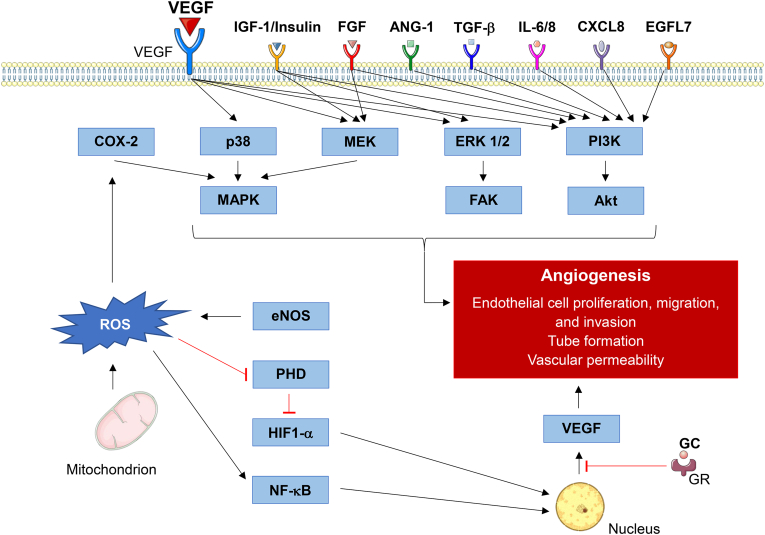
Schematic summary of pathways involved in angiogenesis. This figure offers the reader an overview of the mechanisms involved in transition metals' effects on angiogenesis discussed in this review article. Many of these pathways remain proposed theories, which have yet to be fully understood.

Angiogenesis is a complex process; therefore, many pathways are responsible for its modulation. In Fig. 1 we offer an overview of currently established signaling pathways, growth factors and receptors involved in modulating angiogenesis. Direct or indirect modulation of these mechanisms by a transition metal was considered a criterion for the corresponding publication to be included in this review.

## 3 Discussion

### 3.1 Scandium

No relevant studies regarding scandium's effects on angiogenesis were found during the literature search.

### 3.2 Titanium

When searching the terms “titanium in angiogenesis” two hundred and sixty-six articles appeared. Of these, fourteen fulfilled the inclusion criteria and were used in this review. There has been a previous three-part review conducted on the role of several inorganic trace elements in angiogenesis [[9], [10], [11]]. In the third part amongst others titanium is discussed [11]. One study mentioned in the review demonstrated how the topography of titanium surfaces influenced the secretion of proangiogenic factors by osteoblasts via integrin mediated pathways [12]. Integrins are expressed by endothelial cells (EC) and essentially initiate intracellular signaling pathways. These pathways regulate the expression of growth factors, such as the vascular endothelial growth factor (VEGF) receptor, which in turn stimulate angiogenesis [13] (Fig. 1). The expression of these integrins is dependent of the physicochemical characteristics of biomaterial surfaces. Therefore, the composition of biomaterials used as implants can indirectly take part in promoting or inhibiting angiogenesis. This interaction between cells and implant materials is deterministic for the subsequent response of the host tissue [12]. More specifically, after implantation the surface of the implant absorbs different molecules such as proteins and lipids from its surroundings [14]. The composition of these molecules determines the reactions of surrounding cells, for example whether they proliferate or adhere [15]. Several molecules such as extracellular matrix (ECM) proteins and integrins are responsible for the adhesion of cells onto implant surfaces [16].

A further study mentioned in the review compared a polyether-ether-ketone (PEEK) alloy with a titanium alloy, both materials used in orthopedic surgical procedures [11,17]. Osteoblasts were cultured on the different materials and then their microenvironment was analyzed, displaying a significant increase of VEGF-A, fibroblast growth factor (FGF)-2 and angiopoietin (ANG)-1 production on the titanium alloy as opposed to the PEEK (Fig. 1). In addition, the rough titanium alloy had higher levels of proangiogenic growth factors than the corresponding smooth alloys [17]. This phenomenon has been observed by other researchers as well [18]. Similar results were found whilst studying endothelial progenitor cells (EPC). These cells expressed a higher amount of VEGF on titanium surfaces then on plastic surfaces coated with fibronectin [19]. More specifically, the sand-blasted and acid etched hydrophilic titanium surface gave way to the highest VEGF production. Sandblasting describes the process of roughing surfaces via a pressurized stream of sand, in other words an abrasive blasting.

Since the review [11], further investigations have been made which support these findings. Osteoblasts expressed more integrin-α-1 after being cultured on microrough titanium surfaces [20]. When integrin α-1 was inhibited, osteoblasts secreted less VEGF-A. Bovine coronary artery ECs also displayed enhanced effects on roughened surfaces [21]. The cells proliferated and adhered better than the control in addition to secreting more endothelial nitric oxide synthase (eNOS) and VEGF-receptor (VEGFR) [21]. Yet it remains unclear, how exactly the microstructure of titanium surfaces leads to increased production of angiogenesis-inducing factors [12]. It should also be acknowledged that the described effects cannot be entirely linked to titanium, hence, there is a need to differentiate between effects linked solely to the physical alterations of the surfaces, and effects induced directly by titanium. Yet even in the studies focusing on the surface properties, the smooth titanium-based materials were more angiogenic than the plastic controls [12,18,20,22,23]. Titanium is commonly used as an implant material in dentistry due to its ideal physical characteristics such as corrosion resistance and mechanical strength [24,25]. Although these implants prove to be biocompatible, there is lacking knowledge on the potential direct effects of titanium on host tissue. Titanium has been thought to be inert, yet recent findings refute this idea, suggesting that implants could release titanium particles into the peri-implant microenvironment and modulate surrounding cells [23,26]. Precisely this release has been linked to the processes used to create the desirable modulated titanium surfaces mentioned previously [21]. Due to the extensive altercations to the implant's surface, NPs can be released which can potentially enter the bloodstream [23,26]. To further investigate these effects, researchers exposed human umbilical vein ECs (HUVEC) to a titanium solution to analyze molecular mechanisms [25]. The medium was produced through the incubation of titanium dioxide (TiO_2_)-based alloys in cell culture medium. Endothelial cell (EC) proliferation constitutes one of the basic mechanisms needed for successful angiogenesis [27]. Indeed, the titanium-enriched medium stimulated the ECs to upregulate various proangiogenic genes including *VEGFR-1*, *VEGFR-2*, *eNOS* and *inducible NOS* (*iNOS*). For example, the production of eNOS leads to (nitric oxide) NO synthesis, an important mediator of the formation and proliferation of endothelial monolayers [28]. Furthermore, the phosphoinositide-3-kinase (PI3K)/protein kinase B (Akt) signaling axis, which mediates several crucial parts in angiogenesis, was upregulated in the cells treated with TiO_2_ containing medium [25]. Not only does the PI3K/Akt pathway result in an increase in VEGF secretion, but it also regulates NO and ANG expression [29] (Fig. 1). This pathway is critical in both physiological vessel formation and tumor angiogenesis, making it interesting for therapeutic approaches. The angiogenic markers hypoxia-inducible factor 1-alpha (HIF1-α) and VEGF were also increased in lung epithelial cells exposed to titanium dioxide nanofibers [30]. In addition, after treatment with TiO_2_, the cells were implanted in xenograft rat models, which led to tumor growth. HIF1-α was measured once again, showing increase in the tumors from the cells treated with TiO_2_ [30].

In addition to being used in dental implants, titanium can also be found in stents used in cardiovascular interventions. In an experiment, cells that were exposed to nickel/titanium (NiTi) NPs displayed higher levels of ANG-4 and HIF1-α [31]. Furthermore, angio-reactors containing NiTi NPs were implanted into mice for fifteen days to evaluate angiogenesis. Angio-reactors presented 1-cm silicon tubes containing Matrigel, which resembles ECM, and the NiTi NPs. The reactors containing NiTi NPs displayed an increased number of ECs. Interestingly, reactors containing solely Ni also had increased ECs, however fewer than the NiTi group. Although Ti was not tested separately, these results indicate that Ti also partook in the stimulation of EC migration. The authors hypothesize that these effects are linked to cytokine releases leading to inflammatory processes [31]. Especially when considering the manufacturing of stents, these are relevant findings. Diseases such as coronary artery disease and arteriosclerosis can lead to the occlusion of blood vessels, which cuts of the blood and therefore oxygen supply of the ensuing tissue. As a result, the tissue suffers from ischemia and can become irreversibly damaged. The purpose of a stent is to reopen the lumen of blood vessels, which have been clogged up by ruptured plaques or blood clots. Unfortunately, a long-term risk of these stents is in-stent re-stenosis [32]. This can be the consequence of neointimal hyperplasia and neo-arteriosclerosis. Both these processes occlude the stent lumen, causing ischemia. When considering the materials used to produce stents, their angiogenic potential could be of vital importance. If the material where to be proangiogenic, it could promote EC proliferation. Like the previously reported risk of neointimal hyperplasia, an increased stimulation of ECs within the stent's lumen could also cause re-narrowing of the blood vessels [33]. Ultimately this could lead to ischemia. Therefore, the angiogenic potential of stent materials should be considered during production to avoid such complications.

The review also sheds light on studies indicating antiangiogenetic properties of titanium once applied in the nanoscale [11]. Titanium dioxide (TiO_2_) nanoparticles (NP) (20 nm) led to inflammation in human bronchial epithelial cells via the production of reactive oxygen species (ROS) [34]. Interestingly, these effects were linked to the scale of the particles, as larger particles (200 nm) did not display oxidative damage. TiO_2_ NPs also led to suppressed neovascularization in murine retinas and VEGFR-2 inhibition in human retinal microvascular ECs [35]. Further studies found similar TiO_2_ induced oxidative stress reactions in neural cells and liver cells [36,37]. Concerning the toxicity of titanium in the form of NPs, a more recent publication investigated potential adverse effects [38]. Using the angiogenesis proteome profiler, an assay using antibodies against various angiogenesis markers, differences between the control and TiO_2_ medium could be detected. Indeed, the TiO_2_ exposure resulted in the inhibition of the angiogenesis promoting molecule persephin and the induction of the antiangiogenic endostatin. Furthermore, the proangiogenic proteins platelet derived growth factor and ANG-2 were elevated in the control arm [38]. As TiO_2_ NPs can be found abundantly in various products from our daily lives such as sunscreen, cosmetics, paint and plastics, potential cytotoxicity and angiogenic effects should be further investigated [38]. It should be noted that the TiO_2_ NPs did not display any cytotoxic effects during the experiments.

There is no reported risk for carcinogenesis when TiO_2_ is ingested by humans, however, nanotoxicity on the reproductive system is inconclusive [39]. Moreover, inhaling TiO_2_ NPs is toxic and particles have been categorized as B2 type carcinogen (inhalation toxicity). It must be emphasized that concentration discrepancies exist in literature, from which threshold the TiO_2_ (nano) particles are toxic. Important to note, that the exposure of TiO_2_ to UV light may lead to experimental inconsistencies in the laboratory, where toxicity tests can be hampered by the photocatalytic activity that is triggered by such irradiation.

**Fig. 2:**
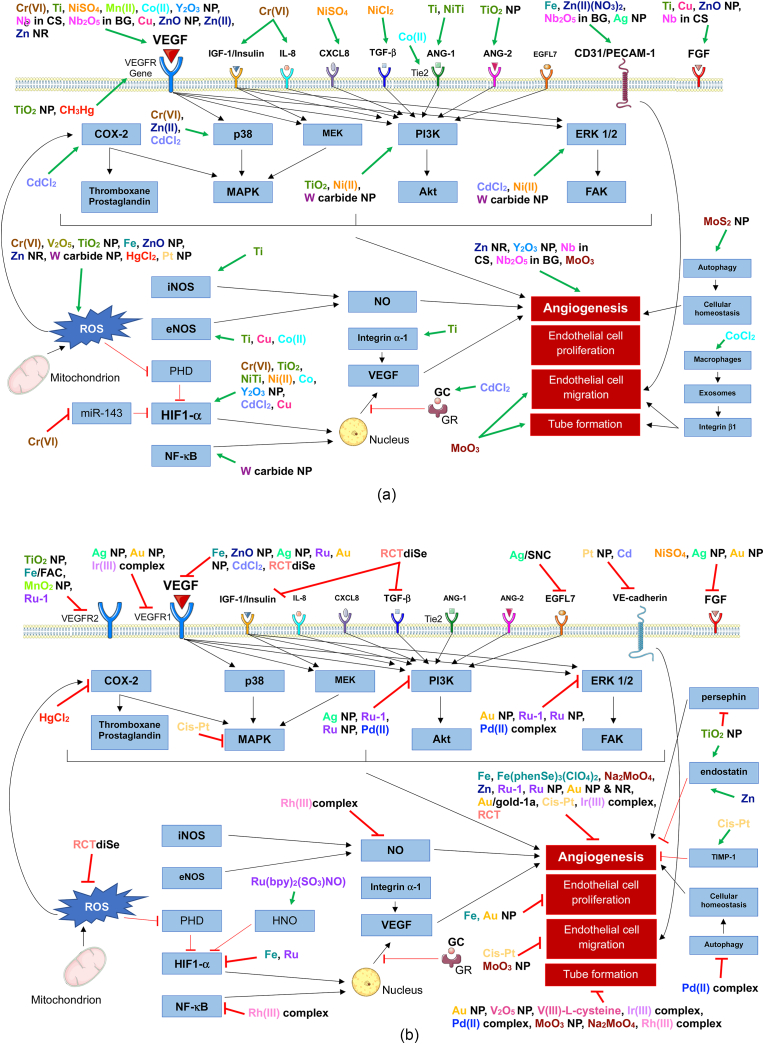
Overview of transition metals activating pro-angiogenic effectors and pathways (A) as well as transition metals inhibiting angiogenesis (B).

In summary, titanium materials provide an overall proangiogenic setting with few exceptions (induction of persephin and endostatin), however, the mechanisms behind titanium's proangiogenic effects require further investigations (Table 1, Table 2 and Fig. 1, Fig. 2B).

**Table 1:**
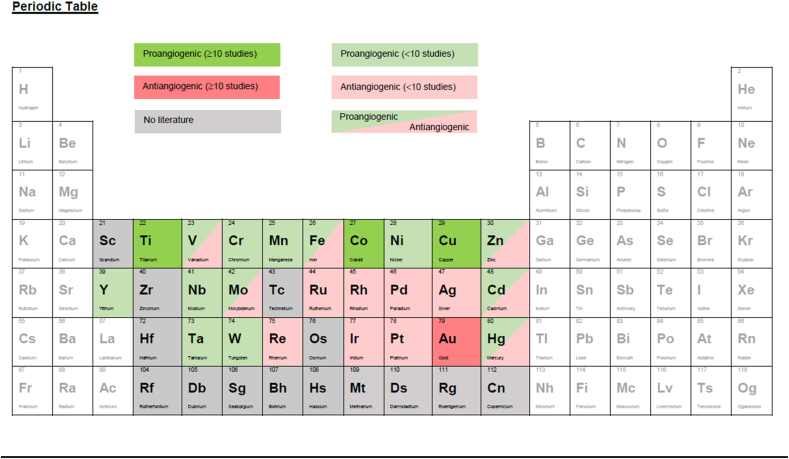
Schematic summary of the angiogenic effects of transition metals. The illustrated color scheme represents a visualization of the number of studies indicating pro- and or antiangiogenic effects. The angiogenic effects of transition metals shaded in grey remain unknown. This table offers an overview of the current findings and should serve as an intuitive way to navigate through this narrative review according to the readers interests.

**Table 2:** Results summarized for titanium and its angiogenic properties.

Titanium species	Experiment	Major results – indicating pro- or antiangiogenic effects	Reference
Unalloyed commercially pure titanium disks	Osteoblasts cultured on titanium disks	Enhanced secretion of angiogenic growth factors by osteoblasts via α_2_β_2_ integrin Conditioned media from osteoblast cells led to EC proliferation	[12]
Titanium alloy (Ti6Al4V)	Osteoblast-like cells (MG-63) cultured on titanium alloy	Enhanced secretion of angiogenic growth factors (VEGF-A, FGF-2, TGF-b, ANG-1)	[17]
Submicron and nanometer titanium surface	Rat aortic ECs and rat aortic smooth muscle cells	Promotion of EC proliferation and long-term intracellular collagen and elastin synthesis	[18]
Sand-blasted, acid-etched titanium disks	EPCs cultured on titanium disks	Enhanced secretion of VEGF	[19]
Microrough titanium surface (coarse grit-blasting)	Osteoblasts cultured on titanium surface	Increased integrin α−1 expression	[20]
Anodized TiO_2_ nanotubes on Ti6Al4V alloy	Bovine coronary artery ECs	Enhanced proliferation, adhesion as well as eNOS and VEGFR secretion	[21]
Titanium surfaces (acid-etched/coarse-grit-blasted)	HUVECs	Enhanced expression of angiogenic factor genes and adhesion molecule genes	[22]
Smooth hydrophobic titanium surface	HUVECs in co-culture with osteoblast-like cells (MG-63)	Proliferation and expression of angiogenesis associated genes in HUVECs	[23]
Titanium-enriched medium	HUVECs treated with medium	PI3K/Akt signaling pathway significantly enhanced Augmented expression of VEGFR1, VEGFR2, VEGF, eNOS, and iNOS genes	[25]
TiO_2_ nanofibers	Lung epithelial cells injected subcutaneously in a xenograft mouse model for tumor development	Increased HIF1-α and VEGF secretion as well as *in vivo* tumor growth	[30]
Ni/Ti NPs	Human ECs and angio-reactors containing Ni/TiNPs implanted into mice	Enhanced expression of ANG-4 and HIF1-α in ECs Increased EC count in angio-reactors	[31]
TiO_2_ NPs	Human bronchial epithelial cells	Cellular inflammation	[34]
TiO_2_ NPs	Human retinal microvascular ECs and retina of C57BL/6 mice	Suppressed VEGF-induced tube formation and migration of ECs In vivo inhibition of neovascularization	[35]
TiO_2_ NPs	Human colorectal adenocarcinoma ECs	Inhibition of persephin and the induction of endostatin	[38]

### 3.3 Vanadium

When searching the terms “vanadium in angiogenesis” thirteen articles appeared. Of these, six fulfilled the inclusion criteria and were used in this review. There has been a previous three-part review conducted on the role of several inorganic trace elements in angiogenesis [[9], [10], [11]]. In the third part, amongst others vanadium is discussed [11]. The review discussed three relevant studies; HUVECS exposed to vanadium pentoxide (V_2_O_5_) resulted in increased ROS production and inhibited proliferation [48]. Using a rat femoral fracture model, the effects of a locally applied organic vanadium salt (VAC) on angiogenesis were studied. After seven days, the researchers noted an increased number of blood vessels and augmented VEGF-C levels within the callus of the VAC-treated rats [49]. Mouse epidermal cells were exposed to vanadate, which led to an increase in VEGF release [50]. At the time no further studies had been conducted concerning vanadium's role in angiogenesis. Since then, there have been further publications that should be considered.

A more recent publication studied V_2_O_5_ NPs and their role as a melanoma treatment in an *in vivo* study [27]. Various cancer cell lines which were treated with V_2_O_5_ NPs displayed cytotoxic effects whereas the non-cancer cell lines did not. Specifically, the HUVECs proliferation was significantly inhibited by the vanadium NPs as opposed to the controls. A vital step in angiogenesis is the formation of tubes [35], so by means of the tube formation assay, this process was analyzed. In the V_2_O_5_ NPs group the HUVECs tube formation was significantly inhibited [27]. A further fundamental step in angiogenesis is the migration of cells to the site of de-novo blood vessel sprouting [51]. By employing the scratch wound healing assay, the NPs were able to reduce the migration process of the HUVECs. The preformed chorioallantoic membrane (CAM) assay displayed damaged and ruptured blood vessel post V_2_O_5_ NP treatment. Additionally, mice bearing melanomas treated with V_2_O_5_ NPs had an increased survival rate as opposed to the untreated controls. Considering these findings, the researchers conclude that vanadium appears to influence several crucial processes involved in angiogenesis in an inhibitory fashion, making it a viable candidate for anticancer treatment [27].

Similar effects have been found in experiments using vanadium oxide nanorods (VONR). As in the previous study, application of the VONRs to the CAM assay resulted in inhibited blood vessel growth [52].

Investigation of vanadium on mice implanted with breast adenocarcinoma cells resulted in further evidence suggesting antiangiogenic effects [53]. Specifically, vanadium (III)-l-cysteine (VC-III) was administered *in vivo*. Mice treated with VC-III had reduced blood vessel sprouting which the researchers linked to a decrease of VEGF-A and metalloproteinase-9 measured in their ascitic fluid.

Looking at the toxicity of vanadium, speciation plays a pivotal role. It has been reported that vanadate V(V) is more toxic than vanadyl V(IV), and inorganic vanadium is more toxic than organometallic complexes [54]. Furthermore, ingestion of vanadium is less toxic than inhalation. Particularly nanosized vanadium compounds have been reported to be more toxic than the bulk forms.

In total three studies discussed used *in vivo* models, resulting in more meaningful data than the studies considering cell cultures. One study indicates a proangiogenic effect of vanadium salts [49], whereas two provide evidence that V_2_O_5_ NPs and VC-III can inhibit angiogenic processes [27,53]. Taking these findings into consideration, vanadium has been proven to be both a pro- and antiangiogenic agent (Table 1 and Figs. 1, 2A/B).

### 3.4 Chromium

When searching the terms “chromium in angiogenesis” thirty-eight articles appeared. Of these, three fulfilled the inclusion criteria and were used in this review. There has been a previous three-part review conducted on the role of several inorganic trace elements in angiogenesis [[9], [10], [11]]. In the second part, amongst others chromium is discussed [10]. There are few publications concerning the direct effects of chromium on angiogenesis, therefore, the review discussed its influence on molecules linked to angiogenic processes [10]. One study analyzed the effects of hexavalent chromium (Cr(VI)) on human prostate carcinoma cells [55]. Via the production of ROS, Cr(VI) led to upregulation of VEGF and HIF1-α (Fig. 1). Moreover, the researchers concluded that the increase of HIF1-α was dependent on the p38 mitogen-activated protein kinase (MAPK) signaling cascade [55] (Fig. 1). In another study, by using an *in vitro* model with nontumorigenic human lung epithelial cells, further links between chromium and angiogenesis were achieved [56]. After having been cultured in a medium containing 1 mM Cr(VI) from sodium dichromate (Na_2_Cr_2_O_7_⋅H_2_O), micro-RNA (miR)-143 expression was significantly reduced, leading to malignancy and increased angiogenesis. These effects were linked to increased insulin-like growth factor (IGF) −1 receptor and insulin receptor substrate-1 (Fig. 1). Also, the proangiogenic factor interleukin (IL) −8 was augmented after Cr(VI) exposure (Fig. 1). However, it remains unclear how chromium represses miR-143 expression [56]. Since the publication of the mentioned review, there has not been a great increase in literature concerning the effects of chromium on angiogenesis [10].

A recent publication supports the results found in the previously mentioned study concerning miR-143 [56,57]. Blood samples from workers exposed to Cr(VI) were analyzed, measuring significantly lower miR-143 levels in contrast to unexposed workers [57]. MicroRNAs have been linked to tumor growth by inhibition of critical signaling pathways, with miR-143 specifically being a tumor suppressor [57,58]. The level of IL-6was increased in the chromium-exposed group (Fig. 1). Indeed, the researchers were able to link the increase of IL-6 to the inhibition of miR-143. Also, it was confirmed that HIF1-α was inhibited by miR-143. To summarize, chromium appears to induce carcinogenic processes by inhibiting the tumor suppressor miR-143, leading to activation of proangiogenic mediators such as HIF1-α and IL-6 [57].

The chromium toxicity in humans has been described to be based on three different mechanisms; (i) the Cr(VI)-induced carcinogenesis, (ii) genomic instability induced by chromium and (iii) epigenetic modification [59]. Underlying this toxicity, Cr(VI) species, such as HCrO_4_^2−^ may easily enter the cells through channels for anionic ion transport. During reduction to Cr(III), ROS are accumulated, and oxidative stress leads to lipid, protein and DNA damage. Besides ROS formation, adduct formation has been reported to be a major toxic mechanism, with Cr(III)-DNA, Cr(III)-protein-DNA adducts and cross-linking. It has been emphasized, however, that human susceptibility towards chromium toxicity is individual, because polymorphism carriers, metabolism, DNA repair, internal antioxidant system as well as external factors (smoking) may be different between different persons.

The current findings are scarce, and the meaningfulness of the results remain limited as the experiments conducted use only cellularmodels. Nonetheless, chromium appears to harbor certain proangiogenic properties that should be explored in further experiments using *in vivo* models (Table 1 and Figs. 1, 2A/B).

### 3.5 Manganese

When searching the terms “manganese in angiogenesis” one hundred and eleven articles appeared. Of these, two fulfilled the inclusion criteria and were used in this review. Manganese belongs to the essential trace elements required in the human body [60]. Manganese in the form of NPs, more specifically albumin-coated manganese dioxide (MnO_2_) NPs, have been able to subdue tumor hypoxia and enhance radiotherapy [61]. One research group investigated fucoidan-MnO_2_-NPs in combination with radiotherapy on a xenograft mouse model [62]. Angiogenic effects were also studied, with results showing that VEGF-mediated phosphorylation of VEGFR2 was reduced to a greater degree by the fucoidan-MnO_2_-NPs than the MnO_2_-NPs. Both fucoidan- and MnO_2_-NPs did not display significant changes regarding angiogenesis, with the fucoidan-MnO_2_-NPs minimally reducing tumor vascularization. The authors conclude that the fucoidan conjugated to the MnO_2_-NPs was responsible for the antiangiogenic effects [62,63]. Human lung cancer-derived ECs exposed to soluble Mn(II) from MnCl_2_ (in tetrahydrate form) displayed an increase in VEGF promoter activity [64]. Furthermore, the inhalation of soluble MnCl_2_-aerosol was linked to the expression of angiogenic genes *in vivo*. The expression levels of VEGF isoforms VEGF_188_ and VEGF_115_, VEGFR-1, endoglin and HIF1-α were increased in mice exposed to Mn(II) [64].

Manganese toxicity has been associated with irreversible dopaminergic dysfunction, leading to symptoms that resemble Parkinson's disease [65]. It has been reported that changes in the neurotransmission evoked by manganese underlie the symptoms rather than neuronal cell loss. Manganese exposure can furthermore lead to alterations in cardiovascular function. While manganese has a short half-life in blood, the half-life in human bone is about 8–9 years. For risk assessment, novel non-invasive techniques are being developed to assess manganese content in human bone. Finally, increased infant mortality has been linked to high levels of manganese in drinking water. Like reported for chromium, also human manganese susceptibility is highly individual, with age, gender, ethnicity, genetics and other conditions, such as chronic liver disease, playing important roles. These findings show proangiogenic effects, however, remain limited in their quantity and overall relevance. Further investigations regarding manganese's angiogenic effects are necessary (Table 1 and Figs. 1, 2A/B).

### 3.6 Iron

When searching the terms “iron in angiogenesis” six hundred and seventy-five articles appeared. Of these, eightfulfilled the inclusion criteria and were used in this review. There has been a previous three-part review conducted on the role of several inorganic trace elements in angiogenesis [[9], [10], [11]]. In the first part, amongst others iron is discussed [9]. Iron is an essential element playing a vital role in several metabolic processes in humans, namely oxygen and electron transport, DNA synthesis and serving as an enzyme cofactor [60]. Both increased and decreased iron quantities are associated with diseases such as iron deficiency anemia and iron overload cataract [66]. The review discusses the role of iron as an anticancerogenic agent [9]. *In vivo* experiments have displayed tumor growth suppression via iron depletion in mice [40]. Iron is as essential to tumor cells as it is to normal cells [41]. Interestingly, the lack of oxygen could make cancer cells promote vascularizationas a response, making them more dependent on angiogenesis. Considering this, researchers investigated the combination of iron depletion with an established antiangiogenic treatment [41]. Mice were fed an iron deficient diet, which led to a suppressed tumor growth as well as enhanced angiogenesis. Subsequently, the mice were treated with bevacizumab (*anti*-VEGF therapy), with the tumor growth being inhibited the strongest in the iron deficient mice. These findings indicatea synergistic effect of iron depletion and antiangiogenic medication. In addition to using such medication, an iron deficient diet could represent a cost-effective way to potentiate available cancer treatments [41].

The connection between iron deficiency and cancer was further investigated in a study regarding breast cancer [42]. Iron uptake was inhibited in human breast cancer cells via transfection with transferrin receptor-1 shRNA. VEGF levels were significantly higher in the iron depleted cells. The cells were also injected into a xenograft mouse model to further investigate angiogenesis. Again, the lack of iron led to the strongest blood vessel growth. VEGF production is mediated by HIF1-α, which levels can be increased by chelating iron with deferoxamine [43,67]. Young women suffering from breast cancer have a higher mortality than older women [42]. Furthermore, iron deficiency is common in young woman [68]. As angiogenesis is crucial for tumor growth, these findings could prove relevant in future breast cancer treatments. Additional research has confirmed this effect, whilst also investigating possible effects of iron accumulation due to menstrual cessation [44]. Overexposure to iron led to increased oxidative stress via the Fenton reaction (Fe (II) + H_2_O_2_ → Fe (III) + OH^−^ + OH·) [69]. As iron induced oxidative stress has been linked to cancer promotion, the researchers believe that both iron deficiency in young women, and iron overload in postmenstrual woman have negative effects in carcinogenesis [44,70].

Tissue hypoxia is a driving force in tumor growth, creating a proangiogenic stimulus with increased cellular levels of HIF [45]. Iron is a regulating cofactor of prolyl hydroxylase (PHD), an enzyme responsible for keeping HIF expression low during normal oxygen supply [45] (Fig. 1). During hypoxia, intracellular iron levels are low, resulting in HIF stabilization [71]. This is partly due to the downregulation of the GTPase Dynamin-2, as it takes part in iron uptake via transferrin endocytosis [72]. Researchers therefore hypothesized whether this proangiogenic effect of iron uptake inhibition could be reversed though increased iron uptake [45]. Ferric Ammonium Citrate (FAC) is an iron salt that can enter cells passively by avoiding the transferrin linked endocytosis [73]. Quiescent HUVECs were not affected by FAC exposure [45]. After being stimulated with VEGF-A, FAC exposure subsequently led to a concentration dependent inhibition of HUVEC proliferation. To ensure these effects were linked to iron, Deferasirox was used as an iron chelator, reversing the FAC linked inhibition of HUVEC proliferation. Furthermore, FAC treated ECs displayed a decrease of tube length and branch points in a tube-forming assay. VEGF-A treated cells displayed a decrease in VEGFR-2 phosphorylation when exposed to FAC, with further downstream signaling molecules subsequently not being activated. To study iron's effects on angiogenesis *in vivo,* a basement membrane matrix was mixed with Lewis Carcinoma cells and implanted subcutaneously in mice. The experimental group was treated with FAC injections for two weeks. Histological analyses showed no difference in tumor weight between the control and the FAC mice, yet there was a significant decrease in vessel length and vessel nodes in the FAC group. Interestingly, next to all these findings, the FAC did not display any effects on HIF expression. The authors link this to cell-lineage differences and the various cofactors regulating PHD activity [45]. Up until this point, the studies discussed linked iron depletion to the amelioration of angiogenic processes. Yet there is also data indicating that the addition of iron leads to antiangiogenic effects. A novel iron (II)-complex with the addition of selenium (Fe(phenSe)_3_(ClO_4_)_2_ led to the inhibition of VEGF-induced HUVEC proliferation [46]. Notably the control complex only containing iron also had antiangiogenic effects, however, not as strong as the selenium-enhanced complex [46].

Apart from these antiangiogenic effects, there is also evidence to be considered, which indicates iron to have proangiogenic properties (Table 3). Primary Hemochromatosis is a hereditary disease leading to toxic iron accumulation all throughout the body due to excessive gastrointestinal uptake [74]. Specifically, increased iron deposits could harm the retina through ferritin mediated neovascularization [75]. Retinal neovascularization is also found at the end stages of proliferative diabetic retinopathy, ultimately leading to vision loss, therefore, representing a very relevant pathology [76]. Ferritin is a blood protein which stores iron in a non-toxic manner and is therefore, critical for the iron homeostasis in the body [77]. Cleaved high molecular weight kininogen (HKa) is an angiogenesis inhibitor produced by ECs [78]. Ferritin can bind to HKa rendering it ineffective. Therefore, ferritin harbors proangiogenic and antioxidant properties [79].

**Table 3:** Results summarized for iron and its angiogenic properties.

Iron species	Experiment	Major results – indicating pro- or anti-angiogenic effects	Reference
Low iron diet	Mice injected with tumor cells	Iron deficient mice displayed inhibited tumor growth	[40]
Iron-deficient diet and an iron chelator	Mice injected with tumor cells, treated with bevacizumab	Synergistic effect of iron depletion and antiangiogenic medication	[41]
Transferrin receptor-1 shRNA	Iron deficient human breast cancer MDA-MB-231 cells injected into mice	VEGF levels were significantly higher in the iron depleted cells and blood vessel growth was enhanced *in vivo*	[42]
Iron depletion by deferoxamine	Sprague-Dawley rats	Increased HIF-1α expression	[43]
Iron-deficient and iron-overloaded diets	Mouse model	Low iron led to promotion of VEGF by stabilizing hypoxia-inducible factor-1α High iron levels increase oxidative stress	[44]
Ferric Ammonium Citrate-Treatment	VEGF-stimulated HUVECs Lewis Carcinoma cells implanted subcutaneously in mice	Inhibition of HUVEC proliferation *In vivo* tumor growth inhibition, significant decrease in vessel length and vessel nodes	[45]
(Fe(phenSe)_3_(ClO_4_)_2_	VEGF-stimulated HUVECs	Inhibition of cellular proliferation	[46]
Conditioned medium from hADSCs treated with an iron-based nanocluster	Mice with ligated femoral arteries	Less tissue necrosis and more frequent limb salvation	[47]

Human adipose derived stem cells (hADSCs) have been used as a proangiogenic treatment in ischemia [80]. Due to limitations, efforts have been made to enhance this therapy with the use of NPs [81,82]. As NPs come with their own adverse effects, a recent study investigated a novel strategy using conditioned medium from hADSCs treated with an iron-based nanocluster (ps-TNC) [47]. The degradation of ps-TNC was developed to be selective to a low pH, specifically degrading within endosomes after endocytosis. ps-TNCs were produced with sodium borohydride (NaBH_4_), polyvinylpyrrolidone (PVP) stabilizer, HAuCl_4_ and FeCl_3_. During dissolution testing, iron ions dissolved from the ps-TNCs under acidic conditions with a higher reactivity with hydrogen. The gold in the ps-TNCs displayed a lower reactivity and its chemical structure remained stable. After concentration optimization, hADSCs were exposed to ps-TNCs resulting in significant upregulation of *HIF1-α*, *VEGF* and *basic-FGF* (*bFGF*). Furthermore, ANG-2, artemin and IL-8 were detected in the ps-TNC-medium using a human angiogenesis antibody array. This medium was then injected into mice with ligated femoral arteries representing ischemic lesions. Albeit limb loss remaining frequent, the mice treated with medium displayed significant differences to the untreated controls, displaying less necrosis and more frequent limb salvation. The authors conclude that using conditioned medium collected from hADSCs exposed to ps-TNC could prove a promising alternative to classic stem cell treatments. The injection of this medium in ischemic lesions could aid angiogenic responses of the host tissue [47].

The toxicity of excessive iron is based on the generation of radicals that damage macromolecules and can lead to tissue injury [83]. Iron overload may lead to hereditary hemochromatosis or accumulation in the liver, possibly resulting in hepatocellular cancer, although the specific mechanism for iron-induced neoplastic transformations are poorly understood. Furthermore, several neuro-degenerative disorders have been associated with changes in iron metabolism in the central nervous system, such as Parkinson's and Alzheimer's disease. Generally, the underlying mechanism for iron toxicity is attributed to the Fenton reaction, where iron catalyzes the generation of hydroxyl radicals and other ROS [84].

Angiogenesis is a dynamic equilibrium between pro- and antiangiogenic factors [46]. Iron and proteins associated to iron such as ferritin, have displayed effects on both pro- and antiangiogenic factors, making it more ambiguous to angiogenesis than other transition metals. This makes iron a very versatile element with potential applications in various diseases, targeting both upregulation as well as inhibition of blood vessel formation (Table 1 and 3 and Fig. 1, Fig. 2B).

### 3.7 Cobalt

When searching the terms “cobalt in angiogenesis” two hundred and seventy-seven articles appeared. Of these, twelve fulfilled the inclusion criteria and were used in this review. Cobalt is an essential trace element in humans and partakes in various physiological processes [60,85]. It is an integral part of vitamin B12, a cofactor for various metalloproteins in the body and is needed for erythrocyte production [85,86]. Cobalt has been used in implant materials in areas such as orthopedics due to its ideal mechanical characteristics [87]. Furthermore, cobalt is known to induce a hypoxic environment through HIF1-α stabilization leading to VEGF upregulation [[88], [89], [90]]. (Fig. 1). Chronic hypoxia and its effects on kidney tissue has been a hot topic for researchers studying renal pathologies, as the kidneys are very sensitive to hypoxic injuries [91]. Often, chronic renal diseases end in microvascular insufficiency, the consequence of which is lacking blood flow [92]. Therefore, promoting angiogenesis could reduce renal damage and prevent further functional loss [93]. Using the remnant rat model, displaying glomerular and systemic hypertension as well as hypoxia, the angiogenic effects of cobalt were investigated [92]. Cobalt chloride (CoCl_2_) dissolved in phosphate buffered saline was injected subcutaneously to ensure a slower distribution rate with no detectable adverse effects. The cobalt-treated rat displayed an increase in HIF regulated genes, including *VEGF*, *Erythropoietin* and *Tie2*. Also, cobalt influenced the healing process of the tubulointerstitial injuries, with the cortical region displaying less tubular atrophy and fibrosis than in the control remnant rats. Antibodies targeting microvascular ECs identified capillaries around the glomeruli. Indeed, the exposure to cobalt led to the restoration of capillaries within the damaged kidneys [92].

As previously discussed, hypoxia plays a key role in the stimulation of angiogenesis. Specifically, hypoxia can stimulate macrophages which have been found to be present during the remodeling of microvascular networks [94]. In addition, macrophages can secrete VEGF-A [95], a trait which enablestumor macrophages to promotetumorigenesis [96]. Therefore, targeting macrophages poses a therapeutic window to influencing vessel growth. Researchers studied the effects of cobalt on macrophages and the ensuing angiogenic potential [97]. The focus was on the exosomes secreted from the macrophages. These exosomes contain various biomolecules such as RNAs, which can modulate processes like angiogenesis [98]. Murine macrophages were treated with a CoCl_2_⋅H_2_O solution [97]. After being derived, the exosomes from the cobalt treated macrophages were added to ECs. This led to enhanced migration and increased expression of *VEGF* and *eNOS*. 10.13039/100014337Furthermore, *in vitro* and *in vivo* experiments supported the proangiogenic effects of the exosomes. With the use of the tube formation assay, ECs treated with the exosomes displayed an increased tube formation and node count as opposed to the controls. Matrigel plugs containing the exosomes were inserted into mice to evaluate the vascularization process. The macroscopic and histological analysis corroborated with the *in vitro* results. To investigate the mechanisms behind these effects, the researchers measured VEGF levels in the exosomes. Interestingly, there was no significant difference in the VEGF amount between exosome treated with cobalt and the control exosomes, implying a VEGF independent mechanism. Integrins have been shown to stimulate angiogenesis through the promotion of EC migration and overall survival [99]. Integrin β1 expression was the highest in the cobalt-exosome treated ECs, which could be a mechanism of the proangiogenic effects. These results underline the immunomodulatory role exosomes play in angiogenesis and how cobalt can enhance this process [97].

Chemotherapy is a common treatment method to suppress cancer, yet it can alsoinflict damage to surrounding healthy tissue [100]. After the application of chemotherapy, tissue recovery is a vital process that should ideally be enhanced by the anticancer therapy. Using cobalt nanowires in combination with the chemotherapeutic doxorubicin, angiogenesis was promoted after the induced cytotoxic effects [101]. Nanowires were chosen over cobalt NPs, as they can maintain magnetic properties, which are essential for precise drug delivery to the cancer sites and hyperthermia induction. The release of cobalt ions (Co^2+^) led to proangiogenic effects in HUVECs, such as enhanced cell growth, HIF1-α and VEGF expression [101]. Other studies have confirmed these results [102,103]. The addition of cobalt to chemotherapeutics offers a way to kill off cancer cells while simultaneously achieving a proangiogenic microenvironment to promote tissue repair [101].

Due to these promising properties, cobalt has also been integrated in bioactive glasses used in tissue engineering to aid in the vascularization process which remains a limiting factor [104]. Indeed, the integration of CoCl_2_ in the biomaterials increased angiogenesis. The results corroborated with the previously mentioned findings and further studies [105,106]. Through the use of similar experimental techniques using HUVECs and the *in vitro* angiogenesis assay, the researchers found enhanced *VEGF-A* and *HIF1-α* gene expression in addition to advanced tubal formation in the cobalt treated groups [104].

As with various NPs, toxicity remains a topic of discussion, and researchers also underline the possible adverse effects of cobalt NPs [107]. Cobalt-chromium based materials, commonly used in hip implants due to favorable characteristics, have been shown to release small particles over time [108]. As discussed beforehand with titanium, this wear-induced release of nanoscale cobalt particles can have adverse effects once they enter the blood stream resulting in cellular damage [109]. This damage is linked to oxidative stress from cobalt exposure. Indeed, cobalt can stimulate HIF1-α under normoxic conditions, which can result in adverse reactions including apoptosis [107]. Specifically, cobalt NPs display higher toxic effects than CoCl2 [110]. Interestingly, the toxic effects of cobalt NPs in HUVECs could be reduced through Fe^2+^ containing molecules, namely ferrous lactate [Fe(CH_3_CHOHCOO)_2_] and ferrous succinate [Fe(CH_2_COO)_2_] [107].

With regard to cobalt toxicity, cobalt bound to serum proteins does not seem to have toxic effects, while free Co^2+^ ion depicts the toxic species [111]. Systemic Co toxicity presents as neurological, cardiovascular and endocrine symptoms. According to corresponding dose-response curves, at concentrations lower than 300 μg/L, such symptoms are reversible, while concentrations higher than 700 μg/L have been associated with severe and irreversible symptoms. As for carcinogenic effects upon inhalation of Co containing dusts, Co has been suggested not to be the main causative for lung cancer, but rather in combination with W carbide compounds, so Co/W carbide was denoted as highly probable for human lung cancer.

Cobalt clearly displays proangiogenic properties, with three of the mentioned studies providing strong evidence from *in vivo* studies [92,97,104]. The toxic effects found also need to be taken into consideration in further studies to achieve safe application (Table 1 and Figs. 1, 2A/B).

### 3.8 Nickel

When searching the terms “nickel in angiogenesis” ninety-nine articles appeared. Of these, eight fulfilled the inclusion criteria and were used in this review. The inhalation of nickel compounds has been linked to the induction of nasal, sinus and lung cancer [112]. Humans can be exposed to different metallic nickel particles through welding fumes during industrial production, though research indicates that not all nickel compounds induce cancer [113]. Specifically, rats which inhaled nickel subsulfide (Ni_3_S_2_) and nickel oxide (NiO) particles displayed tumor growth whilst inhalation of soluble NiSO_4_ 6H_2_O aerosols did not [114]. The authors propose these effects are linked to the water insoluble Ni_3_S_2_ and NiO particles being more readily phagocytized [114]. Next to the chemical characteristics, physical traits such as size have also been shown to be determining for nickel's effects. Indeed, NiO NPs displayed greater cytotoxic effects to murine lung tissue than the micrometer sized NiO did [115]. Further research considering particle size exposed human lung ECs to NiO NPs derived from nickel chloride (NiCl_2_) and both metallic nickel NPs and micron-sized particles [116]. After intracellular uptake, 50% of the NiO NPs were released as nickel ions, whereas approximately 3% were released from the metallic nickel NPs. The micron-sized metallic particles released the least amount of soluble nickel [116]. HIF1-α was activated the most by the NPs and less by the microparticles [116]. These results corroborate previous findings that nickel can stabilize HIF1-α [117,118].

Chemokine ligand 8 (CXCL8) is a potent angiogenic molecule which stimulates ECs and has been shown to be promoted by NiSO_4_ [119,120]. In a further study NiSO_4_ was administered to human lung fibroblasts [117]. The nickel exposure led to an increase in ANG-like 4, leptin and VEGF in addition to increasing CXCL 1, 3, 6 and 8 (Fig. 1). In contrast, FGF-1 was decreased [117].

Other experiments targeted specific signaling transduction factors to elucidate the possible mechanisms behind nickel-induced VEGF expression. Mouse epidermal cells were treated with NiCl_2_, Ni_3_S_2_ or nickel sulfide all leading to VEGF expression [121]. Inhibitors of the MAPK kinase (MEK) 1/2-extracellular signal-regulated kinase (ERK) pathway and of p38 kinase were used to pretreat the cells before nickel exposure (Fig. 1). The results showed a MEK1/2-ERK, but not p38 kinase dependance of nickel-induced VEGF expression. Also, calcium signaling and PI3K were found to partake in the enhanced VEGF expression [121]. Further research corroborates these results, noting that AMP-activated protein kinase suppression increases NiCl_2_ bound VEGF induction [122]. NiCl_2_ has been shown to augment the expression of VEGF-A via integrin β1 [123]. Integrin β1 in turn can activate transforming growth factor-beta (TGF-β), a pro-fibrotic and cancerogenic cytokine [124]. Hence, nickel may be involved in tumor angiogenesis and metastasis through VEGF-A/TGF-β pathway [123] (Fig. 1).

The toxic effects of Ni for humans becomes manifest in cardiovascular and kidney diseases, lung fibrosis, lung and nasal cancer as well as allergies [125]. The main mechanism behind nickel's toxicity lies in mitochondrial dysfunction and oxidative stress. The Ni^2+^ species may induce apoptosis via mitochondria and caspase. Furthermore, epigenetic alterations have been uncovered after nickel exposure.

Overall, nickel compounds display stimulating effects on multiple stages involved in angiogenesis. However, the studies discussed used only *in vitro*/cellular models. Therefore, the evidence presented remains limited. Further investigations using *in vivo* models would solidify nickel's role in angiogenesis (Table 1 and Fig. 1, Fig. 2A/B).

### 3.9 Copper

When searching the terms “copper in angiogenesis” six hundred and sixty-six articles appeared. Of these, eighteen were included in this review. There has been a previous three-part review conducted on the role of several inorganic trace elements in angiogenesis [[9], [10], [11]]. In the second part, amongst others copper is discussed [10]. The review clearly outlines various research underlining copper's role as a proangiogenic transition metal [10,126,127].

Copper is an essential trace element needed by all living organisms [60,128]. Various enzymes are copper dependent, catalyzing vital processes such as oxidative phosphorylation, antioxidant defenses, blood coagulation and angiogenesis [129]. The copper bound stimulation of angiogenesis has specifically been detected in tumors, as quantitative and histochemical analyses measured higher copper contents in tumor tissues than healthy tissues [127,130]. Copper can promote angiogenesis by targeting various proangiogenic pathways. Indeed, copper promotes the stimulation of ECs to migrate and proliferate [131], in addition toinducing VEGF expression [132,133]. The lack of copper has led to inferior angiogenic responses in rabbit models [134]. These findings concerning copper's proangiogenic effects have led scientists to develop treatments to inhibit copper in cancers through chelation to inhibit vascularization and metastasis [129,135]. For example molybdenum in tetrathiomolybdate (TTM) has been proven to chelate copper, resulting in antiangiogenic effects [136]. This makes TTM interesting not only as a cancer treatment but also incopper mediated pathologies such as Wilson disease [137].

In contrast, stimulating angiogenesis through copper supplementation has been proven to be effective in areas where vessel growth is a desired effect such as tissue regeneration and wound healing [138,139]. Tripeptide copper complexes promote neovascularization, ECM production and the formation of granulation tissue [140,141].

Oxidants such as hydrogen peroxide (H_2_O_2_) can induce VEGF and can be produced via the copper catalyzed Haber-Weiss reaction [142,143]. Keratinocytes treated with H_2_O_2_ alone and H_2_O_2_ in combination with copper sulfate displayed enhanced VEGF expression. However, the latter group induced significantly higher results [139]. As these effects were diminished through the application of copper chelators, researchers conclude that copper mediates the H_2_O_2_ induced VEGF expression [139]. Nevertheless, there have been studies conducted that question the consensus that copper's proangiogenic effects are mediated via VEGF [132,144,145]. Copper sulfide applied to HUVECs had a proangiogenic effect at a concentration of 5 μM, without augmenting VEGF production [145]. Interestingly, higher concentrations of copper resulted in the cessation of the proangiogenic effects, yet increased VEGF production. This would suggest that a mere VEGF overexpression is not enough to enhance angiogenesis [145]. The treatment of 5 μM copper in combination with *anti*-VEGF led to a complete inhibition of angiogenesis. The authors stress that this should not be interpreted as copper promoting angiogenesis via VEGF. Rather these results confirm that VEGF remains paramount in the angiogenesis process.

Copper modulates angiogenesis by targeting other factors including HIF1-α, Angiogenin, FGF-1 and eNOS [[144], [145], [146], [147]]. Specifically, eNOS can modulate VEGF-induced angiogenesis, as was shown in a study where VEGF stimulation in eNOS deficient mice resulted in inferior angiogenic responses [148] (Fig. 1). Other research suggests eNOS could also act as a VEGF independent molecule. An *in vitro* experiment exposed HUVECs to copper sulfate, which resulted in increased cell growth [145]. The chelation of copper reduced these effects, with this inhibition being reversible through further copper exposure. Copper treatment also led to an increase in eNOS, but not VEGF mRNA. Through eNOS inhibition, the reversible effect of additional copper in chelator treated cells was silenced. Furthermore, *anti*-VEGF antibodies did not inhibit copper's induction of cell growth. In other words, the findings demonstrate how copper's effects were eNOS dependent and VEGF independent. These findings seem to apply to copper's effects under *in vitro* conditions using vascular EC cultures, whereas copper effects in larger *in vivo* models are linked to VEGF [144,145]. Indeed, this demonstrates how complex the molecular mechanism can be, and why fully understanding the mechanisms behind copper's effects is indispensable for adequate therapeutic application.

Several signal transduction molecules have been identified which partake in the molecular mechanisms of copper's proangiogenic effects, including protein tyrosine kinase and MAPK [139,149]. Antioxidant molecules for example glutathione (GSH) have the opposite effect, decreasing copper induced VEGF expression [139]. FGF-2 has been augmented through oxidants such as H_2_O_2_ and superoxide ions [150,151]. Indeed, it is plausible that copper can indirectly induce angiogenesis by catalyzing the reaction of proangiogenic oxidants.

The field of tissue engineering, more specifically bone tissue engineering, has started applying biomaterials for treating bone defects, as current methods face limitations and a rising demand [152]. Insufficient vasculature is one of the bottleneck problems remaining, keeping the field from establishing a greater clinical application [153]. To augment vascular responses, the addition of different metal particles in scaffolds materials has been tested. Specifically copper oxide in the nanometer scale, i.e., as NPshas showed promising results [146,[154], [155], [156], [157]]. Copper NPs have a bioactive surface and can achieve a more gradual ion release, which is a desirable trait as burst releases of copper have proven to be toxic [157,158]. Concretely, a recent study compared copper sulfate to copper NPs [159]. The copper agents were applied to chick embryos, with both groups promoting angiogenesis compared to the control. However, the copper NPs displayed greater results than copper sulfate. mRNA expression of VEGF-A and FGF-2 were also enhanced by both groups. The NPs once again exhibited a stronger upregulation [159]. Physicochemical characteristics such as size and dosage enhance copper's proangiogenic properties, therefore, exact characterization and testing of copper agents should be conducted to achieve ideal application [158,160,161]. Also, reports of toxicity should not be neglected as certain copper species as for example Cu^2+^ and Cu ^+^ exhibit cytotoxic effects [[162], [163], [164]].

Diseases associated with copper toxicity are Wilson's disease, with an excess of copper leading to neurodegeneration [165]. Mechanistically, copper toxicity has been reported to result in cell death as a consequence of direct binding of copper to lipoylated components of the tricarboxylic acid cycle [166], but also via overexpression of ROS [165]. Moreover, the speciation plays a pivotal role, as for example CuO NPs are more toxic than Cu NPs [163].

All in all, various studies report copper as a proangiogenic element with potential in clinical treatments (Table 1, Table 4 and Fig. 1, Fig. 2B).

**Table 4:** Results summarized for copper and its angiogenic properties.

Copper species	Experiment	Major results – indicating pro- or anti-angiogenic effects	Reference
CuCl_2_, CuSO_4_	Bovine aorta ECs	Enhanced migration	[127]
Copper ions	Corneal pocket assay	Neovasculogenesis	[126]
CuSO_4_	HUVECs	Enhanced proliferation	[131]
6 mg Cu/kg	Mouse model exposed to chronic pressure overload generated by ascending aortic constriction	Increased VEGF, angiogenesis promotion and cardiomyopathy reversal	[132]
Copper sulfate	Neonatal rat cardiomyocytes	Cu-induced reduction in cardiomyocyte hypertrophy was VEGF-dependent	[133]
Copper bound to ceruloplasmin	Rabbit model	Enhanced angiogenesis	[134]
Novel high-affinity membrane-permeant Cu(I) chelator PSP-2	H460 human lung cancer cells on CAM assay	Significant angiosuppression and decrease in tumor weight	[135]
Tetrathiomolybdate	SUM149 inflammatory breast carcinoma cells xenografted into mice	Significantly inhibited the tumor growth	[136]
Tripeptide-copper complex	Dorsal midline wounds in a rabbit model	Enhanced wound healing	[138]
Copper sulfate	Human keratinocytes	Enhanced VEGF expression	[139]
Tripeptide-copper complex glycyl-L-histidyl-l-lysine-Cu	Wound chamber rat model	Increase extracellular matrix accumulation in wounds	[141]
Copper sulfide	Rat aortic rings	Enhanced angiogenesis (mircovessel density)	[144]
Copper sulfate	HUVECs	eNOS-dependent (VEGF-independent) cell growth	[145]
Copper chelator tetraethylenepentamine	HepG2 cells	Reduced HIF1-α binding to the hypoxia-responsive element (HRE) of target genes	[147]
Copper containing mesoporous bioactive glass scaffolds	Human bone marrow stromal cells	Stimulation of VEGF and HIF1-α	[154]
Copper NPs in Carboxymethyl Chitosan/Alginate Scaffolds	Implantation in rat model	Enhanced blood vessel formation within the copper scaffolds	[155]
Copper oxide incorporated in poly-lactic-*co*-glycolic acid amorphous calcium phosphate nanocomposite	Implantation as a chest wall graft in a mouse model	Enhanced graft vascularization and tissue integration	[156]
Copper NPs	CAM assay	Enhanced vascularization	[159]

### 3.10 Zinc

When searching the terms “zinc in angiogenesis” eight hundred and fifty articles appeared. Of these, eight fulfilled the inclusion criteria and were used in this review. There has been a previous three-part review conducted on the role of several inorganic trace elements in angiogenesis [[9], [10], [11]]. In the second part, amongst others zinc is discussed [10]. The review concludes that zinc has suppressive effects on angiogenesis through inhibition of various growth factors in addition to promoting antiangiogenic endostatin [10,[167], [168], [169]].

Zinc is an essential trace element required for various functions within the human body [60]. After iron, zinc is the second-most abundant trace element in humans and the most abundant within cells [170]. Various enzymes involved in anabolic processes such as tissue maintenance, tissue growth and wound healing need zinc as an integral component [170,171]. Furthermore, zinc plays a role in immunological processes, DNA repair and antioxidant defenses [172]. Indeed, zinc deficiency has been linked to tumorigenesis and impaired bone formation [173,174]. Therefore, investigating zinc's effects on angiogenesis has been a topic of interest. The application of zinc silicate nano-hydroxyapatite/collagen (ZS/HA/Col) scaffolds promoted both angiogenesis and bone regeneration [175]. Specifically zinc nitrate hexahydrate (ZN(NO_3_)_2_⋅6H_2_O) and tetraethyl orthosilicate solutions were used to produce zinc nanocrystals. The scaffolds were implanted in an *in vivo* cranial defect rat model, and the resulting histological analyses showed increased blood vessel formation. Platelet EC adhesion molecule-1 (CD31) and VEGF-A quantification confirmed the proangiogenic effects of the zinc containing scaffolds. ZS/HA/Col scaffolds extracts did not influence bone marrow stromal cells (BMSC) or EC migration, nor did they promote EC tube formation. However, when exposed to scaffold/monocyte conditioned media, both processes were enhanced. This effect could be the result of monocytes transformation into tartrate-resistant acid phosphatase (TRAP) cells post zinc exposure. TRAP cells can release various cytokines such as TGF-β1 and VEGF-A which in turn promote angiogenesis by recruiting BMSCs and ECs to defect sites. These results suggest that monocytes prove to be vital in zinc induced angiogenesis via p38/MAPK pathway [175] (Fig. 1).

In another study, human dermal fibroblast cells (HDF_4_) were exposed to Zn(II) ions and zinc oxide (ZnO) nanorods (NRs) [176]. The NRs were produced with zinc acetate dihydrate (Zn(CH_3_COO)_2_ 2H_2_O). ROS and VEGF levels were measured in HDF_4_ after Zn(II) and ZnO NRs exposure, both being increased in the NR group. Furthermore, the CAM assay was preformed, in which the NRs lead to increased vessel length and size compared to the Zn(II) group. Also, an *in vivo* mouse model was used to further study zinc's angiogenic potential. Indeed, the ZnO NRs enhanced the wound healing process compared to the controls [176]. In a similar study, ZnO NPs incorporated in electrospun polycaprolactone scaffolds also displayed proangiogenic effects [177]. Adult human dermal fibroblast cells were grown on the scaffolds containing ZnO NPs, which led to enhanced proliferation rates and increased expression of FGF-2 and VEGF-A. The highest proliferation was observed in the scaffolds containing 2 wt% ZnO NPs, whereas FGF-2 and VEGF-A levels were highest in the 4 wt% group. The number of branching points in the CAM assay was highest in the 1 wt% scaffolds. In addition, the 1 wt% scaffolds were implanted in guinea pigs for *in vivo* testing, displaying fibroblast and pericyte migration, which marks the initiation of vascular sprouting [177]. The authors believe the proangiogenic effects to be mediated by the ROS-dependent growth factor stimulation discussed earlier [[178], [179], [180]] (Fig. 1).

Zinc deficiency can result in the impairment of organ development, as well as the vascular system [181]. Mice with surgically induced hind limb ischemia subjected to a zinc-deficient diet displayed restricted revascularization [182]. In comparison to the control, the zinc deficient mice displayed decreased perfusion rates and VEGF-A levels in addition to having increased ROS levels. Using a nicotinamide adenine dinucleotide phosphate oxidase inhibitor, the impaired angiogenesis within the zinc deficient mice could be partly restored. Also, the authors examined zinc levels in patients with chronic limb-threating ischemia. To examine a possible correlation, the skin prefusion pressure (SPP) was measured and used as an index for tissue blood prefusion. Indeed, a multiple linear regression analysis indicated a positive correlation between serum zinc levels and SPP [182].

However, there have also been experiments conducted exposing cells directly to ZnO NPs, which resulted in antiangiogenic effects. Researchers tested NP concentrations from 1 to 50 μg/ml where cytotoxic effects started at 35 μg/ml [183]. HUVECs exposed directly to ZnO NPs at sub-cytotoxic concentrations displayed lower VEGF secretion rates and impaired capillary tube formation [183]. These differing results may have to do with the difference in application and concentrations. Through the dissolution of ZnO NPs into Zn^2+^ ions, excess ROS production could be the cause of cytotoxic effects via DNA damage [184,185]. Incorporation in scaffolds could lead to a more controllable ROS production, which can have angiogenic effects as previously discussed [186].

If zinc is ingested highly above the daily recommended amounts of 15 mg/day, with 100–300 mg/day, it results in induced copper deficiency with symptoms of anemia and neutropenia, impaired immune function and adverse effects on the ratio of low-density-lipoprotein to high-density-lipoprotein cholesterol [187]. The toxicity of Zn(II) is associated with excess ROS formation, leading to cytotoxic effects caused primarily by DNA damage [184].

In summary the *in vivo* studies presented offer substantial evidence that zinc harbors proangiogenic properties [[175], [176], [177]]. Also, the results indicating zinc levels to correlate with blood perfusion in patients offer further meaningful data. Indeed, there are reports of antiangiogenic traits, however these findings are limited to cellular studies and therefore offer less substantial evidence [183] (Table 1 and Figs. 1, 2A/B).

### 3.11 Yttrium

When searching the terms “yttrium in angiogenesis” forty-nine articles appeared. Of these, one fulfilled the inclusion criteria and was used in this review. Yttrium has found its application in medicine in the form of lasers, superconductors, and biomedical implants [188]. Yttrium isotopes with their unique characteristics are of special interest. ^90^Y is a radiopharmaceutical used to treat cancer via radioembolization [189,190]. ^86^Y is applied in positron emission tomography imaging whilst ^89^Y is used for magnetic resonance imaging [188]. Yttrium NPs have been shown to exert antioxidative properties, protecting nerve cells from oxidative stress by reducing ROS damage [191]. Recently, poly-ε-caprolactone (PCL) scaffolds loaded with yttrium oxide (Y_2_O_3_) NPs were studied to further investigate yttrium's potential role in tissue engineering [192]. Fibroblast and osteoblast seeding on the scaffolds containing the NPs exhibited superior proliferation and viability. To determine the angiogenic traits of the scaffolds, the CAM assay was performed. The blood vessel diameter and the number of capillary junctions in the experimental group was higher in comparison to the controls. Furthermore, after an implantation period of four weeks in a rat model, the results from histological analysis also indicated enhanced vessel formation. The epidermal growth factor receptor (EGFR) plays an important role in wound healing, inducing cell proliferation and migration [191]. mRNA analyses showed an increase in VEGF and EGFR expression in the experimental group, which could be a possible mechanism behind the proangiogenic effects of the Y_2_O_3_ NPs. Since the antioxidative traits of Y_2_O_3_ NPs can result in transient hypoxia, the authors suggest the increase of proangiogenic factors could be linked to the activation of HIF1-α [192,193].

There is low evidence of yttrium toxicity in humans because not many reports have been published so far. However, a toxicity study in rats, with oral exposure to yttrium nitrate at doses of 0, 10, 30 and 90 mg/kg/day for 90 days followed by a recovery period of 4 weeks, revealed no significant changes attributed to yttrium nitrate - where the readouts were mortality, clinical signs, daily food consumption, body weight, urinalysis, hematology, blood coagulation, clinical biochemistry and histopathology, respectively [194].

To our knowledge there is limited research concerning possible angiogenic effects of yttrium. The evidence presented in the *in vivo* study discussed indicate a proangiogenic effect which should be further investigated (Table 1 and Figs. 1, 2A/B).

### 3.12 Zirconium

No relevant studies regarding zirconium's effects on angiogenesis were found during the literature search.

### 3.13 Niobium

When searching the terms “niobium in angiogenesis” five articles appeared. Of these, three fulfilled the inclusion criteria and were used in this review. There has been a previous three-part review conducted on the role of several inorganic trace elements in angiogenesis [[9], [10], [11]]. In the third part, amongst others niobium is discussed [11]. At the time of publication (2016), the authors concluded there was no relevant literature on niobium's angiogenic potential. Since then, there have been relevant publications that should be considered. In comparison to other metals, niobium harbors low cytotoxic effects and has proven to increase mineralization in osteoblasts [195,196]. Niobium is also widely found incorporated in titanium implants [197]. Researchers integrated niobium pentoxide into silicate bioactive glasses (Nb-BG) to test *in vitro* bioactivity and cellular biocompatibility [198]. VEGF expression in BMSCs was measured after incubation in Nb-BGs extracts. The extracts were attained by preincubation of the Nb-BGs in Roswell Park Memorial Institute medium. Indeed, cells expressed higher levels of VEGF once exposed to the Nb-BGs extracts [198]. Similarly, extracts from Nb-BGs within a double-crosslinked gelatin-hyaluronic acid hydrogel (GH)enhanced migration and tube formation of HUVECs [199]. *In vivo* implantation of the GH-Nb-BGs in a rat bone augmentation model resulted in a synergistic stimulation of osteogenesis and angiogenesis. Specifically, immunohistochemical staining for VEGF and Micro-CT analysis displayed an increased number of blood vessels in the experimental group. BG and GH were tested separately which resulted in inferior results as opposed to the treatment with GH-Nb-BGs. Therefore, the authors confirm the proangiogenic effect's to be linked to niobium [199].

In another study, niobium carbide nanosheets were integrated into 3D printed bone-mimetic scaffolds (NBGS) to treat bone defects after bone cancer treatment [200]. HUVECS exposed to the NBGSs behaved similarly to the cells mentioned in the previous study [199]. Expression analyses revealed an increase in *VEGF-B* and *FGF-2*, a possible mechanism leading to the upregulated angiogenesis. In line with these *in vitro* results, *in vivo* implantation led to denser vessel networks. The authors conclude that the ameliorated angiogenesis is likely to have caused the improved osseous regeneration seen in the NBGS group [200].

As for niobium's toxicity, we did not find any references that reported toxic effect of this transition metal.

Although few studies have been performed, two of the discussed studies offer *in vivo* evidence that niobium harbors proangiogenic characteristics [199,200]. Further research is needed to solidify these results (Table 1 and Figs. 1, 2A/B).

### 3.14 Molybdenum

When searching the terms “molybdenum in angiogenesis” seventy-one articles appeared. Of these, three fulfilled the inclusion criteria and were used in this review. Molybdenum is an essential nutrient for various organisms whilst serving as co-factor for enzymes involved in detoxification [60,201]. In the field of medicine, molybdenum oxide has been applied in ablation therapy against cancer cells due to its physicochemical properties [202]. In a recent study, molybdenum trioxide (MoO_3_) NPs cytotoxic effects on cancer cells was investigated [203]. As tumor progression is linked to angiogenesis, the researchers also evaluated possible angiogenic effects. MoO_3_ NPs inhibited EC migration in the scratch wound assay. Furthermore, results from the CAM assay and the chick aortic ring assay displayed antiangiogenic effects [203]. Interestingly, a different study comparing two molybdenum salts found both pro- and antiangiogenic properties [204]. MoO_3_ exerted proangiogenic effects, whilstNa_2_MoO_4_⋅2H_2_O led to a reduction of vessel density [204].

ECs are vital to vascular homeostasis, therefore, EC senescence leads to a deterioration of angiogenic processes, ultimately resulting in cardiovascular diseases [205,206]. Cellular senescence has been linked to ROS-mediated cellular damage [207]. Interestingly, molybdenum disulfide (MoS_2_) NPs have been shown to protect ECs by inhibiting ROS-mediated senescence [208]. Researchers were able to link the protective effects of the NPs to the improvement of autophagic flux, an important protectory cellular process inhibited by ROS. Human aortic ECs and HUVECS pretreated with MoS_2_ NPs were less prone to senescence. Cell cycle arrest related proteins which had initially been higher in the ROS treated cells, displayed lower levels after NPs exposure. Similarly, the capacity of cellular migration and tube formation could be ameliorated through NPs post ROS exposure. It must be noted that the MoS_2_ NP exposure alone did not have any stimulatory effect on these angiogenic processes. Autophagy is crucial to cellular homoeostasis, responsible for recycling and degradation [209]. With special staining methods, the researchers conclude that the anti-senescence effects of the NPs were due to their ability to enhance autophagy [208].

Reports on molybdenum's toxicity to humans have shown that workers in a metal plant with MoO_3_ exposure had respiratory symptoms, accompanied with elevated levels of lymphocytes and neutrophils in their broncho-alveolar lavage [210]. Although performed in the rat and mouse model, exposure of the animals to MoO_3_ and uptake by inhalation, revealed no clinical symptoms, except for a slightly reduced body weight at the highest concentrations (300 mg MoO_3_/m^3^).

The results discussed are limited by their quantity and quality. Few studies indicate molybdenum to have angiogenic effects, relying on cellular *in vitro* experiments. Yet there appears to be evidence indicating potential pro- and angiogenic effects which require further investigation (Table 1 and Figs. 1, 2A/B).

### 3.15 Technetium

No relevant studies regarding technetium's effects on angiogenesis were found during the literature search.

### 3.16 Ruthenium

When searching the terms “ruthenium in angiogenesis” seventy-two articles appeared. Of these, five fulfilled the inclusion criteria and were used in this review. Ruthenium has found various applications in medicine, most notably in cancer treatment [211]. Due to extensive preexisting knowledge and characterization, ruthenium complexes are being tested as antibiotic, antiviral and antiparasitic agents [212]. Concerning antitumor therapy, ruthenium's chemical properties offer advantages that could solve limitations of the widely used platinum derivative cisplatin [213]. Ruthenium complexes exert their cytotoxic effects by intercalating with DNA, increasing oxidative stress, and disrupting mitochondrial membrane potentials [214,215]. The effects of ruthenium in angiogenesis have also been studied. One study investigated the effects of ruthenium derived compound RDC11 on the HIF1-α pathway [216]. RDC11 is an organoruthenium complex with a covalent bond between the ruthenium- and a phenylpyridine carbon atom. HUVECs exposed to RDC11 displayed an inferior capillary network. These results were confirmed with the *in vivo* plug assay, where RCD11 inhibited angiogenesis. Furthermore, the researchers transplanted human colon tumor grafts into mice. Once again, the treatment with RCD11 reduced vascularization of the xenografts in addition to suppressing HIF1-α target genes such as *VEGF* [216]. Interestingly, other findings indicate that ruthenium's antiangiogenic properties are mediated through nitroxyl (HNO) [217]. The reaction of a ruthenium complex [Ru(bpy)_2_(SO_3_)(NO)]^+^ with the biological thiols GSH and N-acetyl-L-cystein led to the production of HNO, which is an inhibitor of HIF1-α [217].

Further antiangiogenic results were attained in experiments conducted using a macrocyclic ruthenium complex [Ru^III^(N_2_O_2_)Cl_2_]Cl (Ru-1) [218]. Ru-1 exposure led to decreased tube formation of ECs and inhibited vascularization in the CAM assay. Moreover, the expression of proangiogenic VEGFR2 and its downstream signaling kinases Akt and ERK were suppressed [219,220]. The researchers also tested two *in vivo* models, with Ru-1 exposure inhibiting vessel formation in zebrafish embryos and suppressing tumor growth in xenografted nude mice [218]. Other ruthenium complexes such as Ru (II)-8-hydroxyquinoline (PQ)-complex as well as ruthenium NPs have led to similar results [221,222]. Akt and ERK are also responsible for bFGF mediated angiogenesis. Both Ru (II)-PQ-complex and ruthenium NPs exposure inhibited bFGF induced phosphorylation of ERK and Akt [221,222] (Fig. 1).

Ruthenium's toxicity has been discussed with respect to the comparison of Ru complexes versus Pt complexes in cancer therapy. It has been reported that Ru complexes are able to damage DNA by a unique DNA binding mechanism that differs from the mechanism of action described for cis-Pt [223]. Hence, Ru complexes are toxic, however, specifically for cancer cells. Otherwise, it has been reported that Ru complexes are generally less toxic for non-cancer tissue compared to Pd and Pt complexes.

In summary, there are not many reports specifically considering ruthenium's role in angiogenesis. However, four of the studies discussed offer substantial *in vivo* evidence indicating a relevant antiangiogenic capacity of ruthenium agents [216,218,221,222] (Table 1 and Fig. 1, Fig. 2A/B).

### 3.17 Rhodium

When searching the terms “rhodium in angiogenesis” five articles appeared. Of these, one fulfilled the inclusion criteria and was used in this review. Rhodium complexes have shown to be of interest in the medical field due to their cytotoxic effects which have been proven effective as anticancer therapeutics [224]. Novel rhodium (III)-complexes have also displayed antiangiogenic properties by inhibiting lipopolysaccharide mediated NO production in macrophages [225]. NO is generated by iNOS via the nuclear factor kappa-light-chain-enhancer of activated B cells (NF-κB) pathway [226]. Interestingly, the rhodium complex led to a downregulation of NF-κB [225]. Furthermore, VEGF stimulated HUVECS exposed to the complex displayed inferior capillary tube formation in comparison to the controls [225]. NO takes on a crucial role in the mediation of angiogenesis by modulating EC survival, proliferation and migration [227]. The inhibition of NO production through exposure to rhodium complexes offers a potential antiangiogenic treatment.

Also rhodium toxicity has been reported in relation to cancer therapy. In a study by Carneiro et al. Rh(II)citrate was found to be effective against cancer cells, while systemic toxicity was negligible [228].

As this is the only study up to date considering rhodium's angiogenic properties, further research is required to solidify these results (Table 1 and Fig. 1, Fig. 2A/B).

### 3.18 Palladium

When searching the terms “palladium in angiogenesis” nineteen articles appeared. Of these, three fulfilled the inclusion criteria and were used in this review. Palladium is commonly used in dental applications and in prostate brachytherapy [229,230]. In addition to studies regarding anticancer treatment, there have also been findings indicating palladium has antiangiogenic effects. Researchers investigated the angiogenic effects of a synthesized palladium (II)-saccharinate-complex of terpyridine (Pd (II)-complex) [231]. Indeed, the Pd (II)-complex significantly disrupted the tubular networks in the Matrigel tube formation assay. Furthermore, these results were confirmed in the CAM assay, with Pd (II)-complex-exposure inhibiting vascularization. Importantly, the authors note that there was neither toxicity nor membrane irritation observed. Therefore, the Pd (II)-complex offers antiangiogenic effects without significant cellular damage [231]. In a more recent study, the same research group investigated the mechanisms behind the Pd (II)-complex's antiangiogenetic effects [232]. Molecular analysis showed that the complex inhibited the VEGFR downstream focal adhesion kinase (FAK)/proto-oncogene tyrosine-protein kinase/Akt/ERK1/2 signaling pathways involved in angiogenic processes. The authors noted that the complex inhibited autophagy, which has been thought to play a vital role in angiogenesis regulation, albeit there being inconsistent results [232].

Interestingly, a recent study investigated several palladacycles using the CAM assay and found both pro- and antiangiogenic effects, with the proangiogenic palladacycles bearing a diphosphine [233].

Palladacycles are organopalladium derivatives with one or more metal-carbon bonds [234]. All the compounds contained one palladium atom, therefore, the differing angiogenic effects are most likely due to the different ligands and not palladium [233].

There is growing evidence of palladium's toxicity, with inorganic Pd species as for example PdCl_2_ being more toxic than organic Pd, such as organometallic complexes. It has been reported that Pd acts via mitochondrial membrane potential collapse and glutathione depletion. The mitochondrial damage in turn increases oxidative stress with more ROS. In addition, also oxidative phosphorylation is disturbed, resulting in lower ADP/ATP levels in the cells [235].

The findings discussed are not numerous and only one study offers an *in vivo* experiment displaying antiangiogenic effects that can be directly linked to palladium [231]. Yet there appears to be a potential antiangiogenic effect of palladium complexes, which should be further evaluated (Table 1 and Fig. 1, Fig. 2A/B).

### 3.19 Silver

When searching the terms “silver in angiogenesis” four hundred and thirty-three articles appeared. Of these, eight fulfilled the inclusion criteria and were used in this review. Silver, for the human body, has no biological role and is considered toxic for lower organisms [236]. Historically, silver has been used in various ways, e.g., silver canisters to store condiments so to keep them from moldering, silver wires used to suture wounds during wars and silver leaves applied to wounds to prevent infections. Today, silver can be found in dentistry as well as pharmaceutical applications due to its antimicrobial and chemotherapeutic effects [236,237]. There have been efforts made to deduce potential angiogenic properties of silver agents. Silver NPs have shown to inhibit VEGF induced cell migration and proliferation of bovine retinal epithelial cells [238]. Furthermore, the NPs blocked the phosphorylation of Akt and increased the level of caspase-3, a key mediator of apoptosis [238]. Another study found silver NPs to inhibit FGF-2-induced angiogenesis [239]. Silver nitrate was reduced with diaminopyridinyl-derivatized heparin to produce the NPs. Both in the CAM assay and the mouse Matrigel plug assay, silver NP exposure inhibited FGF-2-induced angiogenesis [239]. Other studies investigating silver NPs using the CAM assay confirm these results [[240], [241], [242]]. Further research investigating molecular mechanisms found silver NPs to inhibit HIF1-α and VEGFR, as well as their proangiogenic target genes [240,243]. In an experiment conducted on medaka fish embryogenesis, the previous antiangiogenic results were corroborated [244]. Specifically, silver nano-colloid (SNC) was applied to the embryos using epidermal growth factor-like domain 7 (EGFL7) to evaluate angiogenesis. EGFL7 is a crucial marker involved in angiogenic signaling which is produced solely by vascular ECs [245] (Fig. 1). Indeed, the treatment with SNC led to vascular malformation and EGFL7 levels being decreased [244].

Interestingly, a study analyzing PVP-coated silver NPs found them to exhibit proangiogenic properties [246]. In the Matrigel plug assay, the addition of the PVP silver NPs led to an increased vessel formation and EC migration. After NP exposure, the supernatants of ECs were analyzed for angiogenic factors. At concentrations between 0.5 μg/ml and 5 μg/ml the NPs induced the production of VEGF and NO. Furthermore, ERK1/2 expression was increased in addition to FAK, PI3K/Akt and p38 phosphorylation being augmented. To ensure these effects were linked to silver, PVP alone was tested, which did not lead to an increase of VEGF or NO.In addition, the hemoglobin (Hb) concentrations within the Matrigel were measured, with the NPs group having a higher Hb concentration. This increase of Hb was linked to the NPs in a concentration-dependent manner, indicating a higher perfusion rate. Finally, mouse melanoma cells treated with the silver NPs were injected into mice, with the resulting tumors being significantly more vascularized than the controls. The authors acknowledge the previously discussed antiangiogenic traits and note that there were distinct differences between their experimental design, for example the production technique and size of the NPs used. Furthermore, the researchers avoided using angiogenic stimulants, while previous studies studied VEGF-induced angiogenesis. Indeed, they also found the silver NPs to display cytotoxic effects in higher concentrations, which could be a possible mechanism behind the antiangiogenic effects found in previous studies. The authors conclude that these discrepancies need further evaluation in future research [246].

Silver is used as antimicrobial agent and is effective against biofilm formation [247,248]. Silver NPs have a broad spectrum of application due to their fungicidal and antiviral activity upon oxidative dissolution, resulting in Ag^+^ ions. Silver's toxicity lies in its oxidative activity [249], with ROS formation and subsequent cell damage.

It appears silver, in particular silver NPs, have an inhibitory effect on angiogenesis. These findings were confirmed in multiple studies discussed, presenting substantial *in vivo* evidence [[239], [240], [241], [242],^244^]. Nevertheless, the *in vivo* proangiogenic findings should not be overlooked [246] (Table 1 and Fig. 1, Fig. 2A/B).

### 3.20 Cadmium

When searching the terms “cadmium in angiogenesis” fifty-six articles appeared. Of these, seven fulfilled the inclusion criteria and were used in this review. Cadmium belongs to the elements which have found their way into our environment in higher quantities due to mass industrialization [250]. Measurements from blood, urine and hair samples have shown that, after a certain threshold, cadmium can have toxic effects. Therefore, excessive cadmium exposure poses a health threat to humans [250]. Specifically, the role of cadmium toxicity in the reproductive system has been a topic of interest, as studies have found cadmium agents to modulate endometrial angiogenesis, a vital process involved in the menstrual cycle [251,252]. Human endometrial ECs exposed to cadmium chloride (CdCl_2_) showed a decreased expression of *VEGF-A* and *placental growth factor* (PIGF) [251]. These findings are relevant, as disrupted angiogenic processes can lead to bleeding, infertility, abortions and preeclampsia [253]. These results have been confirmed in more recent studies, which found CdCl_2_ exposure to induce vascular injuries in mouse placenta [254,255].

Glucocorticoid receptor (GR) activation via glucocorticoid (GC) has been linked to angiogenesis inhibition [256] (Fig. 1)., Previously researchers found CdCl_2_ to increase active GC in mouse placenta via the protein kinase R-like endoplasmic reticulum (ER) kinase (PERK)/eukaryotic initiation factor-2α, which is coupled to mitochondrial stress [257,258]. The pretreatment of human choriocarcinoma cells with GR silencing RNA reversed cadmium's inhibition of VEGF-A expression [254]. Similarly, the treatment of CdCl_2_-exposed human placental trophoblasts with melatonin, a hormone which can mitigate ER-stress, resulted in reversed VEGF-A inhibition. Through further testing, the authors conclude that CdCl_2_ inhibits placental angiogenesis by inducing the PERK linked GC/GR pathway [254].

Other research found cadmium to inhibit vascular endothelial (VE)-cadherin, a cell adhesion molecule which is integral for successful angiogenesis [259,260]. Immunofluorescent visualization of VE-cadherin in HUVECs indicated a reduction of cellular junctions after treatment with cadmium. Furthermore, human lung microvascular EC (HMVEC) treated with cadmium showed a concentration-related reduction of tubular formation [259].

Further investigations indicate that cadmium's angiogenic modulation is concentration dependant [261]. At CdCl_2_ concentrations of 5 and 10 μM, HUVECs achieved enhanced endothelial tube formation. Yet, at concentrations of 20 μM these effects were reversed. VEGF and HIF1-α secretion was likewise augmented by CdCl_2_ concentrations of 5–10 μM. Through specific inhibition, the researchers found cadmiums VEGF inducing effects to be mediated by MAPKs, namely c-Jun N-terminal kinase, p38 and ERK [261]. In contrast, a more recent study using the same concentration of 20 μM CdCl_2_ found HUVECs VEGF expression to be augmented, leading to enhanced vascularization in the tube formation assay [262]. In the study, the role of miR-101 and its target gene cyclooxygenase (COX) −2 were investigated. Genetic deletion of COX-2 has been found to reduce the production of VEGF in retinal cells [263]. Cadmium exposure led to an increase of COX-2, whilst the inhibition of COX-2 diminished the VEGF mediated proangiogenic effect of cadmium [262]. Further analysis led the researchers to suggest that cadmium's angiogenic effects are mediated by the miR-101/COX-2/VEGF pathway which is ER-stress dependent [262].

Cadmium has also been found to be a human carcinogen [264]. Indeed, cadmium can find its way into the human body through nutrients, drinking water and cigarettes [265]. It can then accumulate in organs such as the liver, kidney and placenta [266]. Lung adenocarcinoma cells (LAC) treated with low-dose CdCl_2_ were found to have upregulated VEGF expression and secretion in addition to having an increase in HIF1-α expression [267]. Furthermore, HUVECs were co-cultured with the media collected from CdCl_2_ treated LACs, which led to significantly increased migration and proliferation. Although the migration, proliferation and apoptosis of the LACs was not influenced by CdCl_2_, these results indicate a proangiogenic effect [267].

Cadmium appears to have paradoxical effects. Whilst cadmium has been proven to be a carcinogen, it could nevertheless delay tumor growth and metastasis by inhibiting cancer-cell induced angiogenesis [268]. In a previous review analyzing cadmium's effects on tumor angiogenesis specifically, it is stressed that varying levels of ROS induced by cadmium can lead to altering effects [269]. Low levels of ROS stress can set off adaptive responses such as cell proliferation, whereas excess oxidative stress ultimately leads to apoptosis [270,271]. The authors of the review conclude that the dose determines the ensuing effects of cadmium [269].

Cadmium is a toxic transition metal, which is taken up by inhalation and smoking. Cadmium is related to lung cancer, to breast, prostate, nasopharynx, pancreas and kidney cancer [272]. Basic mechanism of toxic action is mitochondrial damage, oxidative stress, reduced ATP levels as well as epigenetic changes [273].

The studies discussed indicate that cadmium can modulate angiogenesis. Indeed, two studies offer evidence from *in vivo* studies indicating antiangiogenic effects after cadmium exposure [254,255]. However, the remaining evidence collected resulted from *in vitro* experiments. Additionally, there are also reports on proangiogenic effects. Therefore, further investigations should be made to further evaluate cadmium's angiogenic properties (Table 1 and Fig. 1, Fig. 2A/B).

### 3.21 Hafnium

No relevant studies regarding hafnium's effects on angiogenesis were found during the literature search.

### 3.22 Tantalum

When searching the terms “tantalum in angiogenesis” six articles appeared. Of these, three fulfilled the inclusion criteria and were used in this review. As discussed earlier, titanium is the most prominent material used in orthopedic implants. Yet despite its wide usage, there remain limitations such as low shear strength, the byproducts of corrosion and its elasticity modulus [274]. Recently, tantalum has been proven to be a viable alternative, displaying corrosion resistance, biocompatibility, and high chemical stability [275,276]. Previous research has established that bone regeneration and angiogenesis are strongly linked to one another [277]. Specially, scaffolds designed to promote angiogenesis should achieve mechanical strength in addition to mimicking physiological ECM [278]. Porous tantalum scaffolds with the addition of gelatin NPs were implanted into mice to evaluate angiogenesis [278]. Although there was vessel growth found within the scaffolds, the addition of BMSC-derived ECs to the scaffolds resulted in a significantly higher vessel density. The highest vessel density was found in the tantalum scaffolds that were seeded with both BMSC-derived ECs and BMSCs [278]. In a different study, porous tantalum scaffolds were enhanced with the addition of strontium (Sr) [279]. HUVECs seeded on the Sr tantalum scaffolds displayed more advanced tubular networks, as when seeded on the plain tantalum scaffolds. Furthermore, *VEGF* and *HIF1-α* were higher in the Sr tantalum scaffolds as the controls [279]. Indeed, tantalum facilitates the incorporation of Sr, but the angiogenic potential of these scaffolds must be due to Sr, which has previously shown to increase angiogenesis and osteogenesis [280,281].

Tantalum NPs incorporated in nanofiber PCL membranes induced EPCs to form vessel-like structures in the tube formation assay [282]. The incorporation of magnesium oxide (MgO) NPs within the biomimetic material significantly augmented this effect. Similar results were found in the *in vivo* experiments, where the membranes were subcutaneously implanted in mice. Considering tantalum's chemical stability, few ions were released into the surroundings. The authors indicate that the proangiogenic effects are probably linked to the MgO NPs, which showed the highest ion release [282]. Furthermore, magnesium agents have previously shown inducing effects on angiogenesis [283].

In conclusion, current findings indicate that tantalum is an advantageous material to produce scaffolds which allow for adequate vascularization. Furthermore, tantalum is a viable option to replace titanium scaffolds [284].

In its metallic form, tantalum is inert and does not pose risks to humans [285]. In addition, tantalum oxides have low risk as they exhibit very low solubility. However, in its oxidized form and as halide salts, tantalum has been reported to be slightly toxic [285]. Furthermore, *in vitro* experiments using tantalum particles showed impacts on cellular apoptosis, but *in vivo* local host response was benign, characterized by vital encapsulation of tantalum particles in soft tissue [286].

The three studies discussed offer *in vivo* evidence with proangiogenic results, however, none of the studies linked these effects directly to tantalum. It remains unclear whether tantalum has a direct effect on angiogenesis, hence, investigations solely focusing on tantalum effects are necessary (Table 1 and Fig. 1, Fig. 2A/B).

### 3.23 Tungsten

When searching the terms “tungsten in angiogenesis” ten articles appeared. Of these, two fulfilled the inclusion criteria and were used in this review. Tungsten has previously been applied in various implants used in medicine [287]. However, long term implants might lead to corrosion due to the bodies specific microenvironment, resulting in toxic effects [287]. A more recent review suggests tungsten to be used in the production of fully transient implantable electronic systems [288].

Tungsten carbide-cobalt NPs have been studied to evaluate their angiogenic potential [289]. The NPs were found to induce activator protein-1, NF-κB and VEGF expression in human bronchial ECs (Fig. 1). Molecular analyses indicated these effects to be mediated via ROS, Akt and ERK pathways. The researchers also applied the ECs, which had been initially treated with the NPs, onto the CAM assay. Analysis of the number of vessel branch points indicated a more than fivefold increase in the NP treated cells compared to the controls [289]. It must be mentioned that these effects cannot be linked solely to either tungsten or cobalt, as the study did not test the transition metals individually. Taking this into consideration, these results remain ambiguous.

A study considering zinc tungsten (IV) oxide NPs found them to harbor antiangiogenic properties [290]. Indeed, NP exposure inhibited vessel growth in the CAM assay. Notably, the authors do not discuss in detail whether the antiangiogenic properties result from zinc or tungsten [290]. As discussed, zinc has been found to be antiangiogenic, therefore, the effects cannot be asserted to tungsten for certain.

Inhalation toxicity of tungsten oxide WO_3_nanoparticles has been reported at concentrations exceeding 5 mg/m^3^in a hamster model, where the animals were exposed to this concentration for 4 days [210]. Importantly, tungsten often augments the effects of other toxic stressors, such as Co, in tungsten/cobalt alloys [291] or when W carbide and Co are combined [210].

Research concerning tungsten's angiogenic potential is limited and the discussed evidence does not offer data which can be linked directly to tungsten. More investigations must be made to elucidate theseeffects (Table 1 and Figs. 1, 2A/B).

### 3.24 Rhenium

When searching the terms “rhenium in angiogenesis” nine articles appeared. Of these, two fulfilled the inclusion criteria and were used in this review. Rhenium has found application in the field of oncology, with the high energy beta-emitting rhenium-188 radioisotope serving as a potent therapeutic in nuclear medicine [292,293]. Apart from these anticancer characteristics, rhenium complexes have also shown antiangiogenic traits. Specifically, rhenium (I)-tricarbonyl-complexes (RCT) were found to inhibit proliferation of colorectal carcinoma cells [294]. Zebrafish embryos with labelled ECs exposed to these rhenium complexes displayed significantly inhibited tumor growth, vascularization, and metastasis. The results achieved with the rhenium complexes were superior to those of the anticancer drug cisplatin and the antiangiogenic drug sunitinib. Furthermore, higher doses of the complex did not display significant toxicity. The authors conclude that rhenium (I)-tricarbonyl-complexes offer advantageous characteristics, and further research on human tumor cells should be pursued [294]. In a different study, a similar RCT chelated by a diselenoether ligand (diSe) was investigated [295]. Breast cancer cells exposed to the complex showed significantly decreased proliferation in addition to secreting less VEGF-A, TGF-β and IGF-1. Interestingly this inhibition was specific to cancer cells, while non-cancer cells were less affected. To elucidate the effect of rhenium, the researchers tested the selenium ligand separately, and found its inhibitory effects on cellular proliferation to be weaker. As the ROS levels after complex-exposure were found to be decreased, the authors suggest this to be part of the mechanism behind the complex's effects on VEGF-A [295]. Indeed, previous work has shown rhenium complexes binding to DNA bases, resulting in intrastrand lesions [296]. Since mitochondrial DNA mutations can generate ROS, this could be a further mechanism involved in the antiangiogenic effects [297].

As for the toxicity of rhenium, there is no general statement, however, reports deal with specific rhenium complexes, such as organometallic Re(I) complexes that have been shown to by cytotoxic to cancer cell lines, leading to ROS production and mitochondrial membrane damage [298]. Furthermore, tri-carbonyl Re(I) complexes have been shown to have anti-cancer characteristics without damaging non-cancer cells [294].

The findings concerning rhenium's angiogenic potential are limited, however the results from the *in vivo* study discussed provide evidence that rhenium harbors potential antiangiogenic effects which should be further investigated [294] (Table 1 and Fig. 1, Fig. 2A/B).

### 3.25 Osmium

No relevant studies regarding osmium's effects on angiogenesis were found during the literature search.

### 3.26 Iridium

When searching the terms “iridium in angiogenesis” thirteen articles appeared. Of these, three fulfilled the inclusion criteria and were used in this review. Iridium-192 is a common radionuclide used in brachytherapy due to its short half-life and highly specific activity [299]. Iridium based complexes have also been applied to treat lung cancer by inducing mitochondrial damage, ER dysfunction and increased oxidative stress [300]. Several bioactive octahedral iridium (III)-complexes have shown to inhibit VEGFR-3, a tyrosine kinase integral to angiogenesis [[301], [302], [303]]. In a zebra fish embryo angiogenesis model, an octahedral iridium (III)-complex interfered with blood vessel development [304]. Embryos which had been xenotransplanted with proangiogenic human pancreatic cells also displayed inhibited angiogenesis post iridium treatment [304].

In its metallic form, iridium has been reported not to be toxic, while oxidized iridium as present in diverse organometallic complexes, exhibits cytotoxic effects and is used as anti-cancer agent. It has been reported that anti-cancer iridium(III)complexes are less toxic to non-cancer cells compared to cis-Pt [305], which was also confirmed for di-nuclear iridium complexes [306]. Such findings present a motivation to further develop Ir complexes for cancer therapy.

The evidence concerning iridium's angiogenic effects are limited, however the *in vivo* study discussed indicates an antiangiogenic potential [304]. Further investigations should be made to establish these findings (Table 1 and Fig. 1, Fig. 2A/B).

### 3.27 Platinum

When searching the terms “platinum in angiogenesis” seven hundred eleven articles appeared. Of these, fivefulfilled the inclusion criteria and were used in this review. Platinum has taken on various roles in modern medicine. Not only is platinum found in medical devices such as pacemakers and stents, but it has also found wide usage in cancer therapy [307]. Initially, cisplatin displayed anti-bacterial properties, specifically inhibiting the growth of *Escherichia coli* [308]. Further research found cisplatin also harbored anti-neoplastic traits [309]. Currently, cisplatin is a first-line chemotherapeutic used to treat various cancers [308]. Research has indicated that in addition to cytotoxic effects, platinum also harbors antiangiogenic characteristics [310]. HUVECs treated with conditioned media collected from lung cancer cells exposed to cisplatin displayed inhibited migration and tube formation [311]. Interestingly, when directly exposed to cisplatin, the HUVECs did not display these antiangiogenic effects. In previous work, the researchers found cisplatin to induce tissue inhibitor of matrix metalloproteinase-1 (TIMP-1) secretion from lung cancer cells [312,313]. The use of TIMP-1 silencing RNA reduced the antiangiogenic effects from the conditioned media [311]. Furthermore, molecular analysis found that MAPK pathway was also involved in mediating cisplatin's effects. Notably, the conditioned media from non-cancer cells did not lead to angiogenic inhibition [311].

Angiogenesis is dependent on angiogenic growth factors (AGF) interacting with proangiogenic receptors [314]. Modulating molecules, including heparan sulfate (HS) and HS proteoglycans, keep the neovascularization process in balance by binding to AGFs, proangiogenic receptors as well as angiogenic inhibitors [314]. Polynuclear platinum complexes (PPC) can interact with HS, resulting in angiogenic inhibition [315]. Furthermore, PPC blocks heparinase, an enzyme overexpressed in tumor cells and responsible for releasing angiogenic factors from the ECM [315,316]. In a different experiment, PPCs were used to specifically target tumor vasculature [317]. EC integrins a_v_b_3_ a_v_b_5_ are activated during tumor angiogenesis and can therefore enable cell adhesion and migration [318,319]. The peptides RGD (Arg-Gly-Asp) and NGR (Asn-Gly-Arg) compete with these integrins to bind ECM proteins [320,321]. The researchers conjugated RGD and NGR containing peptides to platinum (IV)-complexes [317]. RGD-tethered platinum (IV)-complexes demonstrated inhibition of cellular proliferation, whereasthe RGD peptides alone did not. Through the addition of specific integrin ligands, the inhibitory effects of the platinum complexes could be reduced, indicating that the integrins indeed were responsible for mediating these effects. These findings have been confirmed in a more recent study and suggest platinum complexes to be used in targeted drug delivery, avoiding the damage to non-cancerous tissue [317,322].

Platinum NPs have also been applied in cancer photothermal therapy [323]. However, as NP treatment disrupts the integrity of ECs, there are concerns that cancer cells could therefore flow into the blood circulation resulting in unintentional metastasis [324]. Researchers investigated whether platinum NP treatment could also lead to tumor metastasis in breast cancer cells [325]. Indeed, platinum NP treatment in HUVECs led to dysfunctional intracellular junctions whilst decreasing the expression of VE-cadherin, epithelial protein lost in neoplasm and vinculin, all of which are important mediatorsof cellular junctions [[326], [327], [328]]. Furthermore, an *in vivo* cancer bearing mouse model showed that platinum NP treatment led to metastasis of injected breast cancer cells [325]. As ROS levels in NP treated cells were higher than the control, the researchers believe oxidative stress to be partly responsible for the impaired cellular functions [325]. As cisplatin has been effectively used as a treatment against breast cancer, these results present interesting evidence [329]. It appears that the application form of platinum is very relevant regarding the desired effects, with platinum NPs proving to be antiangiogenic but also harboring a potential risk of facilitating metastasis.

Cis-Pt has been used successfully to fight against cancer, however, it has side effects, such as long-term ototoxicity exclusively linked to cis-Pt as well as paresthesia in the fingers and toes attributed to residual serum Pt – even 10 years after therapy [330]. As for Pt NPs, their toxicity depends on the size of the particles [331]. While sub-nano sized Pt particles induced inflammation and hepatocyte death, 15 nm sized Pt NPs did not evoke such effects. Oral administration in animal models revealed that Pt NPs lead to oxidative stress, hepatoxicity and nephrotoxicity [331]. Surface-modification of such Pt NPs has been reported to be a promising anti-cancer strategy [332], but carcinogenicity of Pt NPs has not been investigated yet [331].

In summary, the current findings suggest that platinum has antiangiogenic effects. However, the evidence presented is limited as there are only *in vitro* experiments available. The one study discussed that conducted an *in vivo* experiment considered the metastatic potential of platinum NPs, but this cannot be equated with angiogenesis [325]. Nonetheless, the current evidence found is substantial enough to merit further investigations considering platinum as an antiangiogenic agent (Table 1 and Fig. 1, Fig. 2B).

### 3.28 Gold

When searching the terms “gold in angiogenesis” seven hundred four articles appeared. Of these, thirteen were included in this review. There has been a previous three-part review conducted on the role of several inorganic trace elements in angiogenesis [[9], [10], [11]]. In the first part, amongst others gold is discussed [9]. The review clearly outlines various research underlining gold's role as an antiangiogenic transition metal. Since then, further research has been conducted.

In medicine, gold has been applied in various ways including dental prostheses, endovascular stents and in treatments against rheumatoid arthritis [333,334]. Gold agents have also been shown to be viable anticancer agents. For example, a study conducted more than four decades ago found that auranofin inhibited HeLa cells proliferation [335]. As tumor growth is linked to angiogenesis, efforts have been made to elucidate gold's potential angiogenic effects.

Gold NPs have shown to inhibit several processes involved in angiogenesis, including the inhibition of VEGF-165, bFGF and EC proliferation *in vitro* [[336], [337], [338], [339]]. Premature retinopathy due to pathological angiogenesis can lead to blindness in young children [340]. Using a mouse model, gold NP exposure resulted in suppressed angiogenesis, specifically by inhibiting VEGFR-2 autophosphorylation [341]. The mice were initially exposed to a hyperoxic environment, resulting in retinal neovascularization. Thereafter, the gold NPs were injected intravitreously resulting in the inhibition of neovascularization. Molecular analyses of human retina microvascular ECs indicated that the NPs also inhibited the ERK1/2 pathway [341] (Fig. 1). Gold NPs can also be applied for diagnostic and therapeutic purposes [342]. These theragnostic properties of gold NPs result from the enhanced permeability and retention (EPR) effect characteristic to tumor specific environment [343]. Gold NPs can make use of the EPR effect by accumulating, and therefore, directly targeting tumor tissue from within without damaging surrounding healthy tissue. Researchers have made efforts to enhance gold NPs by adding bioactive peptides which facilitate cellular uptake by penetrating cellular membranes [344]. Similarly, *in vitro* experiments investigating gold NPs with the addition of angiogenic peptides resulted in both angiogenesis inhibition and enhancement [[345], [346], [347]]. In one study researchers tested different peptides which were anchored to oligo-ethylene glycol-capped gold NPs [348]. Indeed, the peptide targeting VEGFR1 enhanced angiogenesis, whilst the peptide binding to neuropilin-1 receptor inhibited angiogenesis in the CAM assay [348]. Interestingly, these results indicate that gold's antiangiogenic effects can be counteracted by proangiogenic mediators. This was corroborated in experiments where VEGF-conjugated gold NPs also promoted angiogenesis in wounds of diabetic mice [349].

For ECs to proliferate and migrate they initially need to go through cell division. Eukaryotic cells go through miosis and then ultimately cytokinesis. Polyethelyene glycol gold nanorods were found to inhibit cytokinesis [350]. The staining of human retinal EC cytoskeletons showed many binucleated cells, indicating a successful mitosis but incomplete cytokinesis after exposure to the gold nanorods. As the nanorods did not inhibit VEGF or Annexin A2, the researchers suggest these inhibitory effects to be mediated by the TGF-β pathway, which is responsible for actin assembly within cells. Results from the *in vitro* tube formation assay, aortic ring sprouting assay and *in vivo* mouse models, confirmed the antiangiogenic effects of the nanorods. Notably these nanorods did not induce significant cell toxicity which could be the result ofthe polyethelyene glycol-coating stabilizing the gold NPs [350,351].

In addition to prolonging the life of cancer-bearing mice, gold (III) meso-tetraphenylporphyrin 1a (gold-1a) also inhibited vascularization [352]. Immunohistochemical staining showed that the micro vessel density within the tumor was reduced after gold-1a treatment in addition to *VEGF2* and *stanniocalcin1* expression being downregulated [352]. *Stanniocalcin1* is a biomarker involved in VEGF/VEGFR-2 and ANG signaling pathways [353].

Gold nanoparticles have been reported to exhibit a rather low toxicity [354]. As found for the Pt NPs, also gold NPs’ toxicity depends on their size. When scaling toxicity of NPs, Au NPs have been suggested to be used as reference NPs due to their low toxicity [354]. In contrast, Au NRs were found to be toxic and therefore subject of reducing their toxicity via surface modifications [355]. Furthermore, Au(III) complexes have been shown to be cytotoxic as well as anti-microbial, and different modifications are being tested to reach a good balance between cytotoxicity and antimicrobial action [356].

As the great majority of studies discussed indicate gold to have antiangiogenic properties, the few proangiogenic effects found must be due to the addition of proangiogenic agents and not due to gold itself [[345], [346], [347], [348], [349]] Especially the studies offering *in vivo* data indicating gold's antiangiogenic effects provide substantial data [341,350,352] (Table 1 and Fig. 1, Fig. 2A/B).

### 3.29 Mercury

When searching the terms “mercury in angiogenesis” fifteen articles appeared. Of these, fivewere included in this review. There has been a previous three-part review conducted on the role of several inorganic trace elements in angiogenesis [[9], [10], [11]]. In the third part, amongst others mercury is discussed [11]. At the time, there had not been many studies conducted regarding mercury's effects on angiogenesis. Mercury has been applied in dental amalgam to fill tooth defects, however, its usage has declined in the past years due to arising health concerns [357]. As a result of accumulation in oceans, toxic methylmercury can enter the body through fish consumption [357]. Early research already found methylmercury to inhibit both migration and tube formation of HUVECs [358]. Chick embryos treated with methylmercury developed major neuronal damage, with the cerebral vessel formation also being severely inhibited [359]. Apart from these pathways, methylmercury also modulates the expression of VEGF-related proteins [360]. Minamata disease was caused by methylmercury poisoning from the digestion of contaminated fish, which resulted in extensive cerebral lesions [361]. As VEGF can influence vessel permeability and therefore, indirectly modulate intracerebral edema, researchers studied methylmercury's potential effects on VEGF expression [360,362]. Human brain microvascular ECs treated with methylmercury showed an increase of PIGF, VEGFR-1 and VEGFR-2 whilst the human brain microvascular pericytes secreted more VEGF-A [360]. As these agents partake in the VEGF-pathway promotion, the researchers believe this to be a possible mechanism behind methylmercury's toxic effects on the brain [360].

There has also been a relationship found between workers exposed to mercury and increased atherosclerosis [363]. Rats exposed to mercury (II) chloride (HgCl_2_) displayed endothelial dysfunction within coronary arteries, which was most likely the result of ROS-mediated stress and decreased NO [364]. More recently, the effects of HgCl_2_ on vascular smooth muscle cells (VSMC) have been investigated [365]. Specifically, VSMCs displayed enhanced proliferation whilst their cell size decreased. Molecular analyses indicated these effects to be mediated by MAPK, oxidative stress, and COX-2 (Fig. 1). COX-2 converts arachidonic acid into thromboxane and prostaglandin, which are both responsible for the promotion of angiogenesis [366]. Interestingly, the dissected mesenteric arteries from the *in vivo* rat model had a decreased wall thickness [365]. Such vascular remodeling has been linked to the development of cardiovascular diseases, therefore, the researchers suggest mercury to be a relevant risk factor [365].

Toxicity of mercury has been described for HgCl_2_ and methylmercury ((CH_3_)_2_Hg), the two most prominent mercury species. While Hg(II) induced ROS formation and oxidative stress as well as cell death, *in vitro* studies with cell cultures and gene expression showed that cell exposure to methylmercury leads to many more changes in gene expression compared to Hg(II) [367] and has therefore to be considered more toxic.

Since the review was published [11], we have found one relevant studies that hasbeen published which discussed methylmercury's modulation of VEGF-related proteins [360]. However, this study used *in vitro* methods studying possible molecular mechanisms and does not offer evidence indicating a direct promotion of vascularization [360]. The three studies discussed providing *in vivo* data of mercury's angiogenic effects offer more substantial evidence [359,364,365]. As two studies indicate antiangiogenic effects [359,364], whereas the third provided proangiogenic evidence [365], mercury appears to have an ambiguous role in angiogenic stimulation. Further research would aid in establishing both effects (Table 1 and Fig. 1, Fig. 2A/B).

### 3.30 Rutherfordium

No relevant studies regarding rutherfordium's effects on angiogenesis were found during the literature search.

### 3.31 Dubnium

No relevant studies regarding dubnium's effects on angiogenesis were found during the literature search.

### 3.32 Seaborgium

No relevant studies regarding seaborgium's effects on angiogenesis were found during the literature search.

### 3.33 Bohrium

No relevant studies regarding bohrium's effects on angiogenesis were found during the literature search.

### 3.34 Hassium

No relevant studies regarding hassium's effects on angiogenesis were found during the literature search.

### 3.35 Meitnerium

No relevant studies regarding meitnerium's effects on angiogenesis were found during the literature search.

### 3.36 Darmstadtium

No relevant studies regarding darmstadtium's effects on angiogenesis were found during the literature search.

### 3.37 Roentgenium

No relevant studies regarding roentgenium's effects on angiogenesis were found during the literature search.

### 3.38 Copernicium

No relevant studies regarding copernicium's effects on angiogenesis were found during the literature search.

### 3.39 Comparison

One basic mechanism of pro-angiogenic stimulation lies in the formation of ROS that is activated by many transition metals species, such as Cr(VI), V_2_O_5_, TiO_2_ nanoparticles, Fe, ZnO nanoparticles and nanorods, W carbide nanoparticles, HgCl_2_ and Pt nanoparticles, respectively (Fig. 2A).

Subsequent to ROS formation both COX-2 is activated (and downstream effectors like MAPK) as well as NF-κB that in turn stimulates VEGF expression. Moreover, COX-2 can be directly activated by CdCl_2_ and is inhibited through methylmercury.

Obviously, stimulation of VEGF as a key driver of pro-angiogenic pathways has been reported for various transition metals (Cr(VI), Ti, NiSO_4_, Mn(II), Co(II), Y_2_O_3_ nanoparticles, Nb carbide nanosheets, Nb_2_O_3_ in bioglass, Cu, ZnO nanoparticles and nanorods as well as Zn(II)); while others inhibit or downregulate VEGF (Fe, ZnO nanoparticles, Ag nanoparticles, Ru, CdCl_2_, Au nanoparticles, RCT-diSe). Interestingly, transition metal members of the 4th period of the periodic table are involved rather in pro-angiogenic processes, while members of the 6th period rather act anti-angiogenic (Table 1 and Fig. 2). This is also substantiated when the main signalling pathways, i.e. p38/MAPK, ERK1/2/FAK and PI3K/Akt, respectively, are compared for the transition metals. While direct activation of these pathways are reported through the presence of Cr(VI), Ni(II), TiO_2_, Zn(II), Cu (and only CdCl_2_ from the 5th and W carbide nanoparticles where W occurs in the 6th period); all inhibiting and anti-angiogenic transition metals referenced in this review article that are directly inhibiting the corresponding pathways originate from the 6th period (cis-Pt, Ru-1, Ru nanoparticles, Pd, Ag, Ru nanoparticles, Pd (II)) – except for Ag found in the 5th period. We speculate that the size of the transition metals plays a major role in affecting those pathways, with trends of smaller transition metal (ions) being more pro-angiogenic as opposed to larger transition metal (ions) that are more anti-angiogenic.

Interestingly, both Zn(II) and Cd(II) are reported to stimulate angiogenesis via the p38/MAPK pathway, and both provide a d^10^ electron configuration. Moreover, Cu is also reported to stimulate angiogenesis via this signalling pathway, and Cu(I) also exhibits a d^10^ electron configuration. As for Cr(VI) that furthermore activates the p38/MAPK pathway, it exhibits a noble gas electron configuration, very similar to the stabilizing effects of the full d orbitals in Cu(I), Zn(II) and Cd(II), respectively. Such common electron configurations might support the mechanistic processes underlying the activation of the p38/MAPK pathway. However, further in-depth mechanistic studies are needed to substantiate this observation.

## 4 Limitations

As with every work, there are limitations to be considered. Publication bias, especially in a novel and growing field such as transition metals in angiogenesis, should not be overlooked. Positive results can be overstated, whilst negative or neutral findings are not included. In certain cases, the angiogenic effects found in studies could not be directly linked to the according transition metal itself. Furthermore, many studies used differing cell types, concentrations, exposure times and outcome measures. Various results found in publications were qualitative. Therefore, many results found cannot be directly compared to one another, as there is a lack of substantial research using standardized, comparable methods. Consequently, the summary offered in this narrative review does not offer the same powerful conclusions as found in a meta-analysis. This overview should serve as an initial contact point with this research field. It should offer a comprehensible idea of the current state of knowledge to help orientate readers in this novel field and to inspire further research.

## 5 Conclusion

In conclusion, the current findings prove there to be a great potential in the usage of transition metals to modulate the process of angiogenesis. It should not be neglected that this field is in the very early stages, as various studies mention that the underlying mechanisms behind the effects found remain mostly unknown. One study cannot possibly conduct experiments considering various dosages, sizes, and exposure times; hence, the findings must be considered in the right context.

As this review tries to outline, there has already been great efforts made to investigate alternative agents to treat angiogenesis-related diseases. Many promising results which have been found, however, approximately half of all the transition metals have not yet been investigated regarding their potential angiogenic effects. Therefore, we believe it should be of great interest to begin investigating the remaining transition metals, whilst nevertheless further investigating the already known effects.

## Author contributions

The authors declare that this review was written by the authors mentioned in this article.

## Funding

This research did not receive any specific grant from funding agencies in the public, commercial or not-for-profit sectors.

## Ethical approval and consent to participate

This article is a narrative review and does not contain any human or animal studies performed by the authors.

## Declaration of competing interest

The authors declare that they have no known competing financial interests or personal relationships that could have appeared to influence the work reported in this paper.

## Data Availability

No data was used for the research described in the article.
